# Harnessing extracellular vesicles for ischemic stroke management

**DOI:** 10.1093/rb/rbag038

**Published:** 2026-03-05

**Authors:** Khan Haroon, Jie Yu, Renke Li, Chuancheng Ren, Zhaoting Li

**Affiliations:** Department of Neurology, The Second Affiliated Hospital, School of Medicine, The Chinese University of Hong Kong, Shenzhen & Longgang District People’s Hospital of Shenzhen, Shenzhen, Guangdong 518172, China; Department of Pharmaceutical Science, Division of Biomedical Health Sciences, School of Medicine, The Chinese University of Hong Kong, Shenzhen, Guangdong 518172, China; Department of Biomedical Sciences, Division of Biomedical Health Sciences, School of Medicine, The Chinese University of Hong Kong, Shenzhen, Guangdong 518172, China; Department of Pharmaceutical Science, Division of Biomedical Health Sciences, School of Medicine, The Chinese University of Hong Kong, Shenzhen, Guangdong 518172, China; Department of Biomedical Sciences, Division of Biomedical Health Sciences, School of Medicine, The Chinese University of Hong Kong, Shenzhen, Guangdong 518172, China; NMPA Key Laboratory for Research and Evaluation of Pharmaceutical Preparations and Excipients, State Key Laboratory of Natural Medicines, Department of Pharmaceutics, China Pharmaceutical University, Nanjing 210009, China; Department of Biomedical Sciences, Division of Biomedical Health Sciences, School of Medicine, The Chinese University of Hong Kong, Shenzhen, Guangdong 518172, China; Division of Cardiovascular Surgery, Toronto General Hospital Research Institute, University Health Network, Toronto, ON M5G 1L7, Canada; Division of Cardiac Surgery, Department of Surgery, University of Toronto, Toronto, ON M5B 1L7, Canada; Department of Neurology, The Second Affiliated Hospital, School of Medicine, The Chinese University of Hong Kong, Shenzhen & Longgang District People’s Hospital of Shenzhen, Shenzhen, Guangdong 518172, China; Department of Pharmaceutical Science, Division of Biomedical Health Sciences, School of Medicine, The Chinese University of Hong Kong, Shenzhen, Guangdong 518172, China; Department of Biomedical Sciences, Division of Biomedical Health Sciences, School of Medicine, The Chinese University of Hong Kong, Shenzhen, Guangdong 518172, China

**Keywords:** EVs, exosome, ischemic stroke, biomarker, drug delivery, brain

## Abstract

Extracellular vesicles (EVs) are versatile, cell-derived complexes that serve as a complete package of biologically important molecules, released during both normal and pathological processes. With rapid advancements in EVs research, there has been a shift in its therapeutic applications, clinical development and commercialization. EVs have been extensively studied for their potential to mitigate ischemic stroke pathological mechanisms, such as excitotoxicity, immune response, blood–brain barrier (BBB) dysfunction, neuroinflammation and apoptosis. EVs’ innate ability to protect molecular cargo (proteins, DNA or RNA) from enzymatic degradation, their natural ability to traverse BBB, high cargo-loading efficiency and superior biocompatibility make them ideal candidates for targeted drug delivery systems in ischemic stroke. Their presence in most biofluids and changes in their contents during disease conditions, support investigation of EVs as promising minimally invasive biomarkers for the early diagnosis and treatment monitoring of ischemic stroke. Moreover, EVs in combination with other therapeutic agents have considerable potential for clinical translation. This perspective provides a comprehensive overview of current literature on EV-based systems in stroke management, highlighting their role as standalone therapeutics, presenting opportunities for cargo delivery, biomarkers discovery and synergistic component of combination therapies. Additionally, the ongoing clinical trials are discussed along with the challenges and considerations required to advance the clinical translation and adaptation of EV-based systems for ischemic stroke management.

## Graphical Abstract

**Graphical Abstract:**
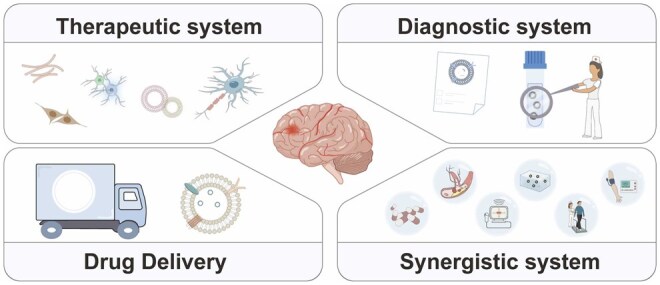

## Introduction

Extracellular vesicles (EVs) are naturally occurring, membrane-derived, heterogeneous nano-sized vesicles enclosed within a phospholipid bilayer. They are secreted by almost all cell types and are present in cell/tissue culture supernatants as well as in various biological fluids including saliva, blood, urine, synovial fluid, cerebrospinal fluid, lymph and breast milk. EVs act as a key “communicator” within the body, playing a vital role in numerous processes, such as (i) trafficking of internalized cargo throughout the body, (ii) disposal of cellular debris, (iii) contributing to the formation of new structures and (iv) facilitate cell signaling and could induce or reduce a pathological condition depending on the type and state of the secreting cells [1, 2].

**Figure 1:**
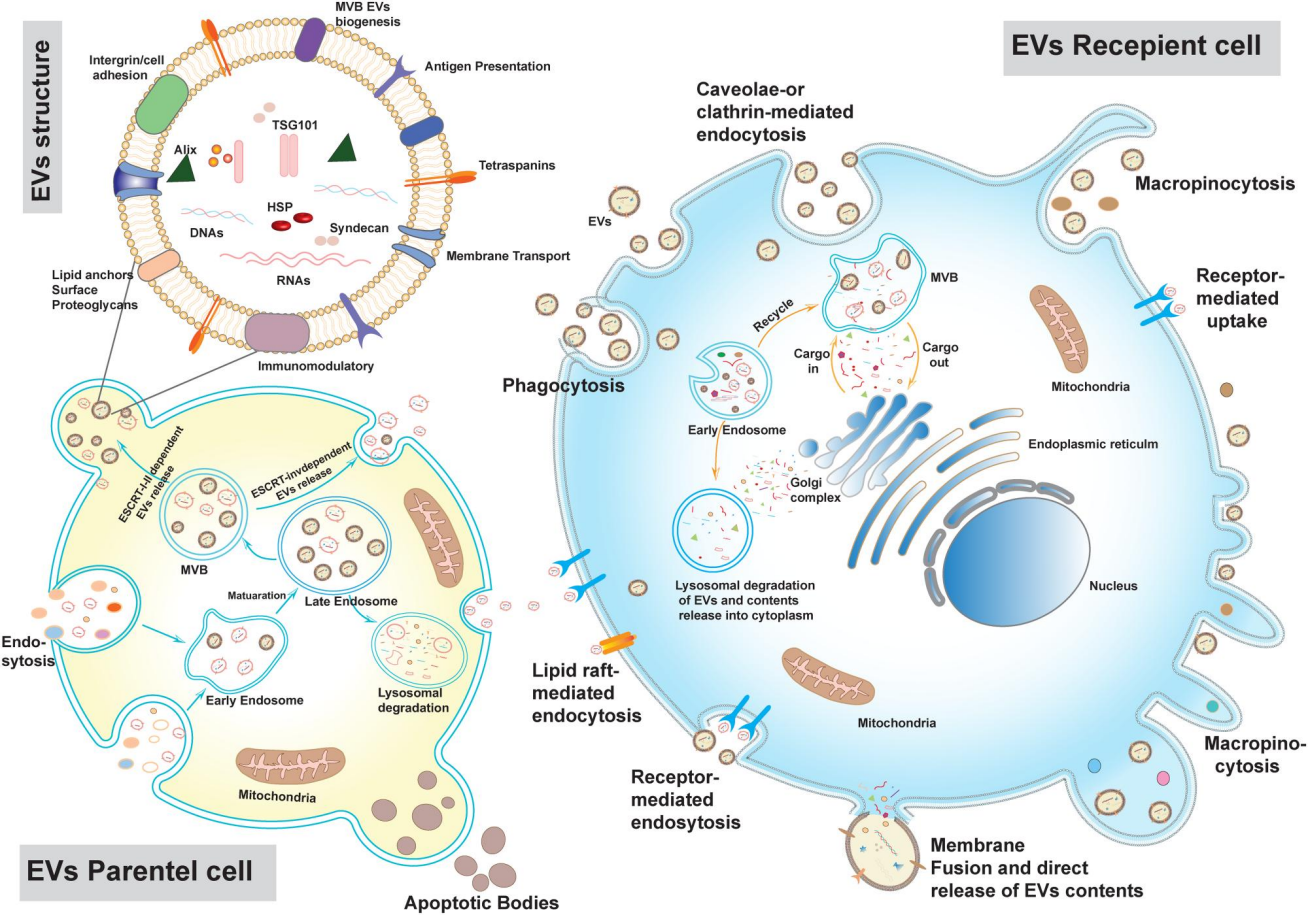
Cell-to-cell exchange system of EVs in living organisms. EVs are generated through the endosomal biogenesis pathway and taken up by the recipient cell through multiple mechanisms. Once the cell uptake EVs, they could release their cargo into the lysosome, be directed to lysosome for degradation or traffic back to endosomal compartments and be recycled to extracellular space.

EVs are formed within cells through the inward budding of the endosomal membrane, which generates intraluminal vesicles that subsequently merge to form multivesicular bodies. Various cellular machineries are involved in regulating these processes including endosomal sorting complexes required for transport (ESCRT) machinery [3]. Key ESCRT-associated proteins such as tumor susceptibility gene 101 (TSG101), heat shock protein 90 and ALG-2-interacting protein X (ALIX), are often present in released EVs. For reliable study outcomes, the identification of EVs typically involves confirming the presence of tetraspanins (CD9, CD63, CD81), ESCRT, source-specific markers, particle number and EV functionality [4]. EVs internalization by target cells has been observed through several mechanisms, including endocytosis, macropinocytosis, phagocytosis, clathrin-coated lipid raft-mediated pathways, receptor-mediated processes and membrane fusion [1, 5, 6]. During endocytosis, the cell membrane folds inward to engulf EVs, a process mediated by proteins such as clathrin or caveolin. Macropinocytosis allows cells to engulf extracellular fluid and EVs in bulk, whereas larger EVs can be internalized by immune cells through phagocytosis. In some cases, EVs fuse directly with the plasma or endosomal membrane of the target cell, releasing their contents into the cytoplasm [7]. Additionally, EVs may interact with specific receptors on target cells, triggering intracellular signaling without the need for internalization [2]. Once internalized, EVs may release their cargo in the cytoplasm, be degraded in lysosomes or be recycled back into the extracellular space, highlighting the versatility of EV-mediated cell-to-cell communication [8] (Figure 1). It has been estimated that EVs are produced at a rate of approximately 100 particles per hour in the circulatory system [9] and around 200 particles per cell under *in vitro* conditions [10].

Although initially considered mere cellular debris, EVs are now recognized as critical mediators of cellular communication, biomolecular transfer, personalized diagnosis and therapy. EVs carry a diverse range of cargo, including proteins, lipids and nucleic acids, which reflect the state of the parental cell [11, 12]. Under normal physiological conditions, EVs facilitate the maintenance of homeostasis and regulate immune responses, whereas during pathological states, EVs can spread harmful cargo, including misfolded proteins, RNAs and oncogenic factors, thereby contributing to disease progression [13, 14].

The blood–brain barrier (BBB) poses a significant challenge in the development of effective therapeutics for ischemic stroke and other neurological disorders. Due to their highly selective permeability, over 98% of small-molecule and biological drugs fail to achieve effective therapeutic concentrations within the brain, limiting their effectiveness. EVs offer a promising solution owing to their ability to cross BBB, small size, high biocompatibility, low immunogenicity, cell-to-cell communication and efficient cargo-loading capabilities [15–17]. These attributes make EVs ideal candidate for therapeutic applications in brain disorders, including stroke. Recently, numerous studies have investigated EVs as cell-free therapeutic agents for stroke. EV-based therapies have shown the potential to mitigate critical processes in stroke recovery, including inhibition of apoptosis, mitochondrial dysfunction, inflammation and promotion of neurogenesis and angiogenesis. EVs derived from stroke patients contain specific cargo, such as microRNAs and proteins, that reflect the underlying damage and inflammatory processes, making them promising candidates for noninvasive biomarkers. This enables earlier and more accurate stroke diagnosis. Furthermore, EVs possess natural intrinsic stability in circulation due to their negatively charged surface and presence of CD47 which trigger a “do not eat me” signal to evade the mononuclear phagocytic system [18]. These natural features, combined with their safety profile and cargo loading efficiency, make EVs an ideal candidate as drug delivery vehicle for ischemic stroke. Ongoing preclinical and clinical research has highlighted their potential as viable alternatives to traditional stem cell therapies, signaling a paradigm shift in ischemic stroke treatment strategies.

This review explores the versatile role of EVs in ischemic stroke, focusing on their potential as therapeutic agents, cargo delivery vehicles, biomarkers and synergistic agents. Additionally, it addresses the challenges and limitations associated with their clinical application and explore strategies to overcome these obstacles. Throughout this review, the term ‘EVs’ is used to refer to vesicles within the 30–200 nm range, as commonly defined in the literature.

## Mechanism of ischemic stroke and role of EVs

Ischemic stroke is a clinical condition of neurological dysfunction caused by cerebral, spinal or retinal cell death due to infarction following vascular occlusion or stenosis. The ischemic stroke hemisphere mainly has two zones: the ischemic core and the ischemic penumbra. The ischemic core consists of damaged cells, whereas the penumbra represents the area surrounding the core, located between normal and infarcted tissue. Most therapeutic approaches target the restoration and protection of the penumbra, although the ischemic infarct core can also be treated depending on the severity and timing of blood reperfusion [19].

Brain cells are critically dependent on the circulation of blood to supply oxygen and glucose, which are essential for their optimal function. During ischemic brain injury, the deprivation of oxygen and glucose leads to insufficient ATP production, resulting in the breakdown of sodium-potassium pumps on the cell membrane. Such disturbances cause an increase in intracellular calcium ions, which in turn triggers the production of pathophysiological agents such as reactive oxygen species (ROS), proteases or phospholipases, ultimately leading to brain cell death, vascular alterations and inflammation [20]. The mechanisms of cell death in ischemic stroke involve several overlapping and interconnected processes, including excitotoxicity and ionic imbalance, immune response/neuroinflammation, mitochondrial dysfunction and apoptotic-like cell death [21–23]. A mounting number of studies have demonstrated EVs’ role in mitigation of ischemic stroke pathologies including excitotoxicity, neuroinflammation, BBB dysfunction, hypoxic injury, angiogenesis and neurogenesis in rodent models [24] (Table 1).

**Table 1:** Native EVs as therapeutic system for ischemic stroke.

EVs source	*In vivo*-administration dose/rout/time	Study model	Techniques used	Therapeutic outcome	Ref.
Embryonic stem cell-EVs	1 × 10^9^ EVs, i.v. injection for 3 days after MCAO	*In vitro* cellular assay/*In vivo* mice/tMCAO	Proteomic, apoptosis, RT-qPCR, flow cytometry, NTA, TEM, WB, neurobehavioral tests, histological stainings	Leukocyte and inflammatory cytokine, infarct volume, long-term neurological deficit ↓ Regulatory T cells, neuronal survival ↑	[25]
M2-Microglia-EVs	(4 μg/kg) EVs I.V injection from Day 1 to Day 7. 100 μg for 3 days after tMCAO	*In vitro* OGD and *in vivo* mice/tMCAO	NTA, transmission electron and confocal microscopy, flow cytometry, *in vivo* and *in vitro* studies, PCR	Recovery of mNSS and ischemic brain injury, ↑ Astrocytes proliferating gene, GFAP, Notch 1 expression, ↓ Sox2 expression	[26, 27]
Adipose-derived stem cells-EVs	I.V injection of 100 μg EVs from 1 to 7 days after tMCAO	*In vitro* OGD and *in vivo* mice/tMCAO	NTA, transmission electron and confocal microscopy, flow cytometry, *in vivo* and *in vitro* studies, PCR	↓ Infarct size and ↑ Neurobehavioral recovery, PTEN and STAT1 expression	[28]
Neural progenitor cells-EVs	10 μg, 100 μg or 1000 μg of EVs systemic administration at Days 1, 3 and 5 after MCAO	*In vitro* OGD and *in vivo* mice/tMCAO	NTA, electron and confocal microscopy, flow cytometry, RT-PCR, proteomics, *in vivo* and *in vitro* studies	↑ Neurological recovery, neuroregeneration and blood B and T lymphocytes, ↓ Cellular death	[29]
Human adipose tissue derived-EVs	Single dose of 200 µg/kg of EVs, intranasal administration 24 h after tMCAO	*In vivo* mice/tMCAO	NTA, Transmission Electron and confocal microscopy, flow cytometry, MCAO, neurobehavioral assessments	BBB reconstruction, re-stabilization of vasculature in peri-infract area, decreased infarct size and neurobehavioral recovery	[30]
MSC derived-EVs	2 × 10^6^ of EVs I.V injection immediately after 30 min of MCAO or 100 µg of EVs 2 h after tMCAO	*In vitro* OGD and *in vivo* mice and rats/tMCAO	TEM, NTA, WB, MCAO, flow cytometry, microscopy, chromatography, immunostaining and immunohistochemistry.	↓ Neurological deficits, infarct volume, brain edema, brain leukocytes and neuronal injury, immunosuppression. ↑ Functional and neurological recovery, neuroregenration, angiogenesis.	[31–35]
M2 microglial-EVs	100 µg of EVs I.V injection for 7 or 2 days after tMCAO	*In vitro* OGD and *in vivo* mice, rats/tMCAO	Cellular assays, qPCR, WB, stainings, neurobehavioral and functional tests, TEM, NTA, flow cytometry	↑ Functional and neurological recovery, White matter repair, BBB integrity ↓ infarct and edema oligodendrogenesis and inhibition of Oligo3 gene.	[36, 37]
NSC-EVs	10 µg EVs+ NSCs lateral ventricle injection on Day 7 after tMCAO	tMCAO	TEM, NTA, WB., MRI, qRT-PCR, Nissl and TTC staining, immunostaining and immunohistochemistry	↓ ROS, Cerebral infarction, Neuronal death and Glial scarring, ↑ Motor function, NSCs differentiation	[38]
M2-EVs	200 µg/mL EVs, tail I.V injection after 2 h after tMCAO	*In vitro* OGD and *in vivo* mice/tMCAO	TEM, NTA, WB, RNA sequencing and pull-down assay, Immunostaining and immunoprecipitation, Neurobehavioral and functional tests,	↓ nerve injury and proptosis, OIP5-AS1 in neuron, MCAO mice and ischemic stroke patients, Infarct volume, ↑ OIP5-AS1 in M2-EVs, TXNIP protein degradation	[39]
Umbilical cord-MSC-EVs	50 μg of EVs, tail vein injection 2 h after occlusion	*In vitro* SH-SY5Y cells OGD and *in vivo* rats/tMCAO	TEM, NTA, WB, qRT-PCR, Cellular transfection, in situ hybridization, ROS, RNA pull-down etc. Neurobehavioral and functional tests	↑ Cell viability, SLC7A11expression, ↓ f erroptosis, infarct volume, neurological deficit	[40]

### Excitotoxicity and role of EVs

Excitotoxicity is a neurotoxic process predominantly mediated by glutamate, the primary neurotransmitter in the mammalian brain. Glutamate plays a key role in neuron-to-neuron communication, brain maturation, synaptic plasticity and neuronal growth. During brain ischemia, the impaired uptake of glutamate leads to its accumulation in the extracellular space, resulting in the overstimulation of glutamate receptors, such as NMDA and AMPA. This overstimulation causes a pathological influx of calcium ions into cells, particularly through extrasynaptic NMDA receptors, damaging cellular structures and impairing mitochondrial function [41, 42]. Overactivation of AMPA receptors also leads to an accumulation of intracellular sodium and chloride ions, disrupting the delicate balance of ions within cells. This triggers water influx into neurons, resulting in cellular and tissue swelling (edema) and eventual cell lysis [43]. Moreover, overstimulation of these receptors could inhibit the P13K/Akt signaling pathway via PTEN activation, further promoting cell death [20]. In the context of excitotoxicity, EVs from various sources have demonstrated the ability to propagate or modulate neurotoxicity through different mechanisms. EVs can mitigate excitotoxicity by transporting anti-excitatory proteins, mRNAs and lipids. EVs from glial cells or neurons could reduce glutamate levels through the transfer of excitatory amino acid transporter 1, which sequesters excess glutamate from the synaptic cleft and extracellular space, preventing overstimulation of NMDA and AMPA receptors [44]. EVs can enhance the intrinsic antioxidant defense of neurons by delivering microRNAs or proteins that upregulate the expression of antioxidants, such as superoxide dismutase and glutathione peroxidase. These enzymes help mitigate ROS production after ischemic stroke [45]. For example, EVs derived from adipose-derived stem cells (ASC-EVs) were found to protect neurons from OGD/R-induced injury *in vitro* and inhibit neuronal apoptosis in a MCAO rat model [46]. Another study demonstrated the neuronal survival potential of EVs after OGD and brain ischemia. The study showed that EVs reduced neuronal death by decreasing autophagic flux and inhibiting p53-BNIP3 activity in neurons. *In vivo*, EVs reduced infarct size and improved neurological recovery in mice. These findings were attributed to EVs containing miR-25-3p [47]. Conversely, EVs could also propagate excitotoxicity by trafficking misfolded proteins, pro-inflammatory cytokines and apoptotic signals [48, 49].

### Immune response and role of EVs

Another critical mechanism in ischemic stroke is the activation of the immune system, which triggers an inflammatory response. Key immune cells in the brain, such as microglia and astrocytes, become activated and release substances that can have both protective and harmful effects. While they help clear cellular debris and promote tissue repair, they also release pro-inflammatory factors that can damage surrounding cells. This inflammation contributes to the breakdown of the BBB, cellular swelling and neuronal damage, all of which exacerbate stroke pathology [19, 50].

EVs have shown promise in counteracting inflammation through their immunomodulatory effects. They modulate immune cell activity and reduce the production of pro-inflammatory cytokines. For instance, MSC-EVs have been shown to reduce infiltration of leukocytes, monocytes and macrophages following stroke, when delivered immediately post-reperfusion or up to 6 h after tMCAO [31]. Similarly, neural stem cell-derived EVs reduced inflammation and increased cellular viability by upregulating ZEB1 expression in OGD/R-treated microglia [51, 52].

In elderly stroke patients, immune system dysregulation is a key factor contributing to higher mortality, increased disability and prolonged hospitalization. Aging is often accompanied by heightened inflammatory stress, as evidenced by elevated levels of pro-inflammatory cytokines, such as TNF-α and increased neutrophil counts in postmortem brain tissue [53–55]. Understanding these mechanisms is critical for improving recovery outcomes in older stroke patients. To address these challenges, MSC-EVs have shown significant promise in modulating the immune response after stroke, particularly in aged mouse models. In a study using MCAO in both young and aged mice, the intravenous administration of EVs improved neuroprotection when delivered immediately or 6 h post-reperfusion. Interestingly, aged mice showed increased leukocyte infiltration but lower counts of macrophages, monocytes and lymphocytes. EVs treatment reversed this trend, decreasing blood monocytes and activating T cells and reducing the neurological deficit, infarct volume, brain edema, neutrophils, inflammation and neuronal injury observed in both aged and young mice groups [31, 35, 56]. MSC-EVs were also found to improve neurological function and neuronal survival by activating the IL-33/ST2 signaling pathway in astrocytes after ischemic stroke [57]. Further research on embryonic stem cells derived EVs has revealed their potential to regulate immune responses post ischemic stroke. EVs therapy significantly reduced white blood cells infiltration, inflammatory cytokines, neuronal death and infarct volume. Additionally, EVs improved long-term neurological function and minimize tissue loss. Mechanistically, these EVs contain elevated levels of TGF-β, Smad 2 and Smad 4 proteins, which activate specific signaling pathways in CD4+ T cells, effectively modulating neuroinflammation and offering protection against ischemic stroke [25]. The immunomodulatory mechanism of EVs in ischemic stroke also involves influencing the activation and maturation of antigen-presenting cells such as dendritic cells (DC) [34]. DC maturation is commonly inferred by upregulation of CD80/83/86, which enhances T-cell priming and adaptive immune activation. MSC-derived EVs have been shown to modulate DC maturation through changes in CD80/86 expression. Stem cells-derived EVs were found to regulate CD40/80/86 expression to restore post-stroke immune balance [58]. Similarly, EVs enriched with miR-146 influenced DC differentiation and expression of surface markers [59]. EVs have also been shown to modulate intracellular signaling pathways essential for DC activation. EVs modulate the Toll like receptor, myeloid differentiation primary response protein 88 (MyD88) and TRIF-dependent pathways, leading to downstream modulation of NF-κB phosphorylation and cytokine production [60, 61]. M2 microglia-derived EVs have shown potential for white matter repair in a tMCAO rodent model, resulting in reduced brain atrophy, enhanced white matter repair, improved mNSS and increased oligodendrogenesis. These effects were attributed to the presence of miR-23a-5p in M2-EVs, which regulated the Olig3 gene, while its knockdown negated the benefits [27].

Ischemic stroke alters T-cell phenotypes over time, including a decline in CD62L+CD127+ memory T cells and an expansion of pro-inflammatory effector subsets, which worsen the recovery [62]. EVs-based therapies have shown promise in modulating immune response after ischemic stroke, but long-term data on memory T-cell persistence remain limited. Most studies report T-cell dynamics at relatively early post-stroke time points (7–45 days), focusing on acute or subacute inflammatory modulation, without extended follow-up to assess the establishment of memory subsets. Embryonic stem cell EVs enhanced regulatory T cells differentiation and neuroprotection for up to 3 weeks, indicating the potential for prolonged adaptive immune modulation through EVs therapy [25]. Sustained infiltration of tissue-resident memory T cells in the chronic post-stroke phase was reported [63], yet functional recall or re-challenge assays were not performed. Future investigation should, thus, include late-phase (≥90 days) phenotyping and functional re-challenge models to clarify whether EVs therapy induce lasting immune reprogramming or transient T-cell activation.

### Mitochondrial dysfunction and role of EVs

Mitochondria, the primary energy-producing organelles, also serve as a major source of oxidative stress production during ischemic-reperfusion injury. Excessive ROS production activates pathways that drive cellular death. Mitochondrial dysfunction during excitotoxicity leads to increased permeability, mitochondrial swelling and membrane collapse, which in turn activates apoptotic and oxidative stress pathways [64]. Mitochondria-containing EVs were found to enhance mitochondrial function, ATP production and brain endothelial cells survival. EVs colocalized with recipient endothelial cell mitochondrial networks, preserving mitochondrial integrity and reducing ischemia-induced damage. Combined with HSP27, these EV mixtures decreased BBB permeability and reduced brain infarct size in a mouse stroke model [65]. Another study compared the efficacy of EVs in restoring mitochondrial function and improving post-stroke outcomes. They found that EVs from mouse brain endothelial cells were more effective in mitochondrial function, enhancing ATP levels and neurological deficit scores, compared to EVs from human brain endothelial cells in a recipient mouse model. These findings indicate that species-matched EVs provide superior mitochondrial recovery and therapeutic benefits in ischemic stroke [66]. Additionally, EVs derived from preconditioned MSCs administered intranasally for the treatment of brain ischemic injury significantly reduced oxidative stress and mitochondrial dysfunction. The therapeutic effects were achieved through the enhancement of nuclear factor erythroid 2-related factor 2 (Nrf2) via a PARK7-dependent pathway. Upon EVs internalization by neurons in the ipsilateral hemisphere, Nrf2 was delivered to the mitochondria, restoring mitochondrial function after brain ischemia [67].

### Apoptotic-like cell death and role of EVs

Apoptosis is a highly regulated, ATP-dependent form of cell death recognized by specific morphological changes, including cellular shrinkage, cytoplasmic condensation, loss of membrane integrity and apoptotic body formation. Neuronal apoptosis is closely involved in post-ischemic stroke pathology. Stroke triggers the intrinsic (mitochondrial) and extrinsic (death receptor) apoptotic pathways, both of which are activated by caspase cascades. Caspases are a family of cysteine aspartases, among which caspase 3 is the most abundant protease in the brain, mainly cleaved in neurons and is present in the ischemic core as well as in the penumbra. Increased caspase activity targets and degrades numerous substrate proteins in several cell compartments, leading to cell demise [20, 22].

EVs from various cellular sources have been reported to protect the cerebral vasculature from apoptosis-like cell death. Rab27a-EVs play a protective role in endothelial cells under ischemic conditions *in vitro* and in a tMCAO mouse model by regulating the caspase-3 pathway. These EVs significantly reduce ROS production and apoptosis by modulating caspase-3 activation [68]. NSC-EVs suppressed apoptosis in ischemic stroke by inhibiting NLRP3 activation and its downstream pyroptotic signaling pathway. EVs reduce the release of inflammatory cytokines (IL-1β and IL-18), preventing caspase-1 activation, thereby alleviating ischemic damage [69]. Another study demonstrated that NSC-EVs prevented cellular death in an OGD-induced injury and mouse stroke model, reducing cell death and promoting neuroprotection by increasing blood B- and T-lymphocyte concentrations [29]. EVs have been shown to attenuate neuronal apoptosis and enhance angiogenesis in brain endothelial cells following OGD/R. The protective role of EVs was largely through the delivery of early growth response proteins, which promoted the expression of SIRT6, a key regulator of cellular stress responses. Early growth response proteins directly bind to the promoter of SIRT6, which in turn inhibits Notch signaling by suppressing Notch1, a pathway known to contribute to cell death and inflammation [70]. Human urine-derived EVs reduced infarct volume, increased neurogenesis and alleviated post-stroke neurological deficits [71]. Additionally, EVs increased axonal-myelin bundle density and enhanced synaptic and dendritic plasticity by downregulating PTEN and deactivating GSK-3β. EVs treatment also enhanced transcallosal axonal plasticity and promoted contralesional axonal sprouting into the denervated spinal cord, aiding the rewiring of spinal motor neurons after stroke [72, 73]. ASC-EVs have also demonstrated neuroprotective potential in stroke recovery [74]. In a tMCAO model, intravenous injection of ASC-EVs 24 h post-stroke significantly improved neuromotor and cognitive functions while reducing atrophy volume. qPCR sequencing identified 14 upregulated miRNAs in EVs, the downstream targets of which were the microglia polarizing proteins STAT1 and PTEN [28].

### BBB dysfunction and role of EVs

The human brain contains approximately 644 km of blood vessels, with about 85% comprising small vessels known as capillaries, which serve as the major site of the BBB. The BBB protects brain cells by preventing harmful factors in the systemic circulation from entering the brain. During ischemic stroke, an abundance of inflammatory factors along with the activation of endothelial cells, macrophages and pericytes leads to BBB disruption. This disruption allows the influx of neurotoxic blood-derived debris, cells and microbial pathogens into the brain, resulting in brain edema [20, 75].

EVs play a crucial role in maintaining BBB integrity after ischemic stroke. iPSC-EVs mitigated BBB damage and reduced leukocyte infiltration and pro-inflammatory factors after brain ischemia. Mechanistically, these EVs activated the eNOS-Sirt1 axis in endothelial cells through AKT1 and CALM, demonstrating a novel therapeutic approach to protect the BBB and improve neurological functions in aged mice [76]. In a comparative study of BMSC-EVs and BEC-EVs for BBB disruption recovery, both types of EVs had the potential to reduce cerebral infarction and BBB leakage and enhance ZO-1 and Claudin-5 expression in a rat stroke model. BMSC-EVs were more effective in suppressing Cav-1 expression, which is implicated in BBB permeability and showed superior neurological functional outcomes [77]. M2 macrophage-derived EVs have been shown to mitigate BBB disruption after ischemic stroke by delivering miR-23a-5p, which targets TNF and modulates the expression of matrix metalloproteases (MMP) 3 and NF-κB p65, thereby preserving BBB stability [37]. After stroke the caveolin-1, CD147 and VEGFR2 pathways become overactive and trigger MMP-2/9 activation. EVs protects the BBB by blocking the signaling pathways that lead to MMP activation [78]. Mice serum derived EVs reduced cell death and preserved the BBB integrity both in living rats and lab-grown brain cells. EVs therapy influences autophagy by altering specific proteins and preventing the degradation of key molecules in brain cells [79].

Furthermore, the risk of hemorrhagic transformation during intravenous thrombolysis with tPA remains a major concern in stroke treatment. MSC-EVs have demonstrated the potential to mitigate tPA-induced BBB disruption by delivering miR-125b-5p to astrocytes and targeting the TLR4/NF-κB pathway to maintain BBB integrity [80]. Moreover, a single intranasal dose of ASC-EVs (200 µg/kg) administered 24 h after pMCAO improved BBB reconstruction and vascular stability in the peri-infarct area, reducing infarct volume. Long-term recovery of motor and behavioral deficits has been observed, highlighting the promising therapeutic effects of EVs in BBB repair and stroke management [30]. In addition to maintaining the BBB integrity, EVs contribute to restoring normal blood flow in the brain following ischemic brain injury [81].

**Figure 2:**
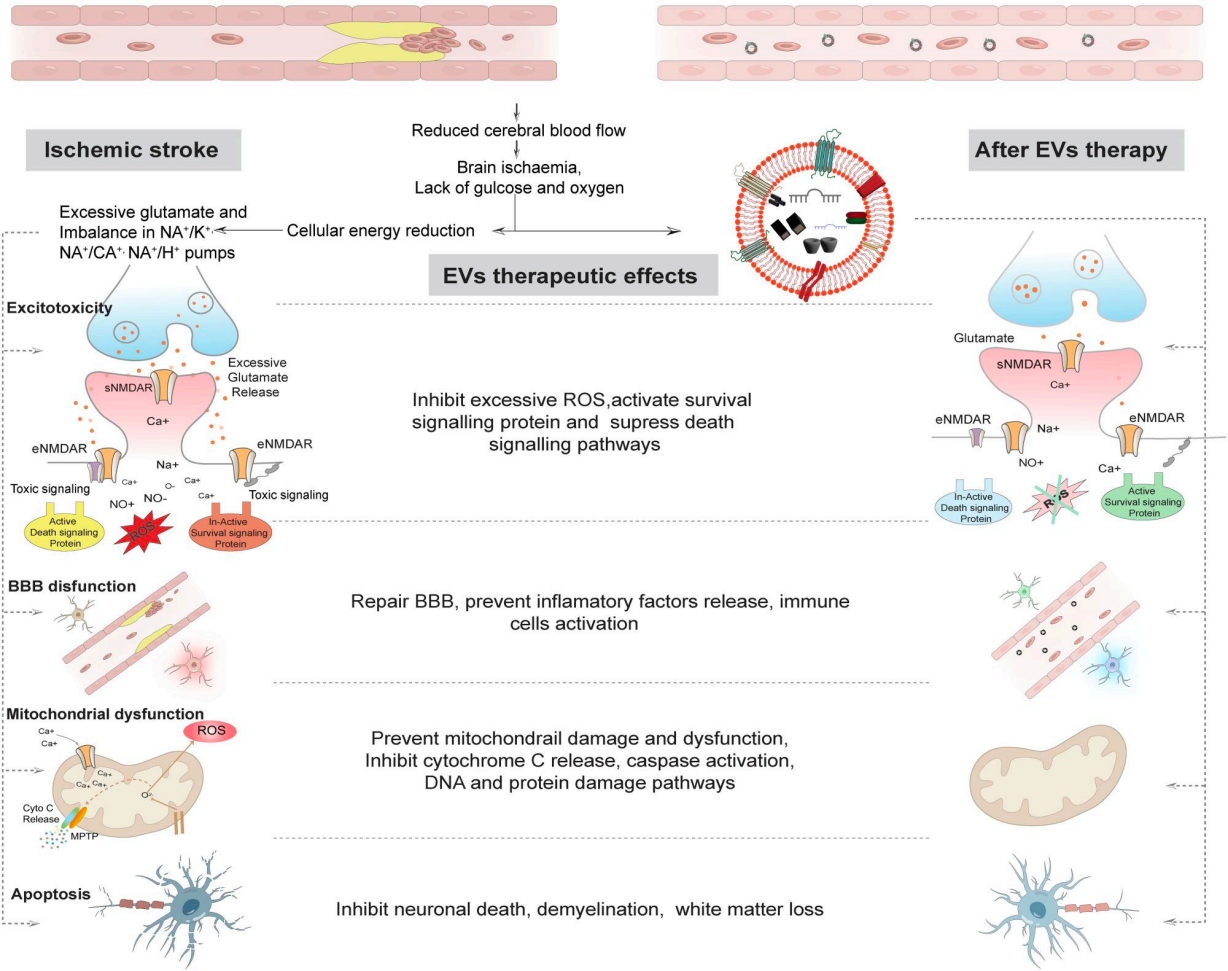
Mechanism of ischemic stroke and role of EVs: Decreased cerebral blood flow because of occlusion of a cerebral artery leads to an OGD environment, resulting in a mismatch between the energy supply and demand in the brain cells. The OGD condition triggers a series of biochemical reactions. (**A**) Excitotoxicity: Energy mismatch causes increased release of glutamate, which leads to overactivation of eNMDARs and subsequent influx of calcium ions into neurons. The over activation of eNMDARs activate toxic signaling and excessive ROS generation. (**B**) BBB dysfunction: Direct damage caused by OGD, which activates immune and inflammatory response. (**C**) Loss of energy also leads to mitochondrial dysfunction. Mitochondria release cytochrome C and active caspase pathways leading to DNA and proteins damage. (**D**) Neuronal death: Autophagy is programmed cellular death while during OGD condition, overactivation of autophagy leads to neuronal death.

Although EVs have been shown to have positive outcomes in stroke and other brain diseases, they have also been shown to exacerbate BBB via the transfer of miRNA that target proteins associated with tight junctions and actin remodeling. Cancer-derived EVs secrete miR-205, which destroys the BBB function of the endothelial monolayer, downregulating the tight junction protein zonula occludens-1[82, 83]. EVs isolated from lipopolysaccharide-challenged mice induced neuroinflammation after injection into normal mice [84]. Stroke patient-derived EVs have an increased inflammatory proteomic profile associated with acute-phase response, including C-reactive protein. When these EVs were applied to human cells, increased expression of tumor necrosis factor mRNA, interleukin-1β mRNA and CXCL-1 mRNA was found, which in turn activated macrophages and induced a proinflammatory response. Stroke patient EVs cell culture treatment also reduced the tight junction proteins ZO-1 and claudin-5 [85, 86]. EVs enriched with miR-124 led to an increase in motor neuron degeneration and neuroinflammation through the production of the inflammatory mediator MMP-9 in mouse microglial cell culture. In addition, EVs derived from Alzheimer’s disease patient brain postmortem tissues were found to accelerate the accumulation of phosphorylated α-synuclein and neuropathological tau protein upon mice intracranial injection [87]. Deriving EVs from postmortem brain tissues opens a new era of exploring the neuropathological role of EVs in brain diseases [88]. However, during EVs sample preparation, risk of contamination from EVs secreted upon cells death must be kept in mind when performing such kind of investigations. Although further investigation is needed to fully elucidate the role of EVs in stroke recovery, existing studies highlight their promising neuroprotective, neurorestorative, ROS mitigation and anti-apoptotic capabilities following stroke-induced brain injury (Figure 2) [89–93].

## EVs-based cargo delivery system for ischemic stroke

Since 2013, when a Nobel Prize in Physiology or Medicine was awarded for the discovery of how EVs deliver cargo between cells, much progress has been made in the field of EVs as cargo delivery systems [94]. The cargo-delivery properties of EVs represent one of the most promising area of study compared to other nanocarriers because of (i) stability in circulation due to negative surface charge, (ii) multifold cargo-encapsulation capacity, (iii) their biological origin, (iv) small size and lipid membrane composition, ideal for fusing with target cells and multiple routes of uptake, and (v) biocompatibility, including their ability to avoid mononuclear phagocytic system and natural biological barrier-crossing abilities. These properties make EVs superior to synthetic nanoparticles, such as liposomes or other nanocarriers [95].

**Figure 3:**
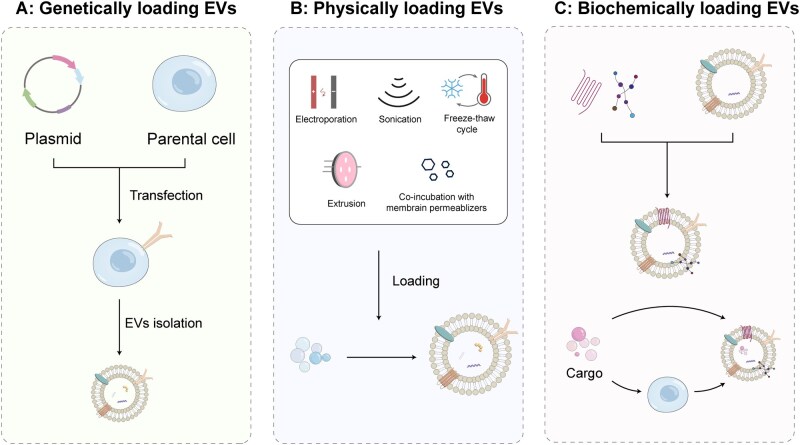
EVs as Cargo delivery system for ischemic stroke. (**A**) Genetically loading EVs: the cargo of interest is overexpressed in EV-producing cell via gene transfection and the cell package the therapeutic payload into their secreted EVs. (**B**) Physically loading EVs: through external physical means the target cargo is incorporated into EVs core. (**C**) Biochemically loading EVs: EVs are functionalized and decorated for specific cargo delivery to target recipient cell.

Recently, much progress has been made in using EVs as cargo delivery systems for stroke therapy (Table 2). Hydrophobic and hydrophilic agents could be easily loaded into EVs core because of the lipid shell structure and the hydrophilic core [109]. Given their natural characteristics for cargo preservation and delivery to target cells, various methods have been developed to load EVs with therapeutic cargo for ischemic stroke (Figure 3).

**Table 2:** EVs-based cargo delivery systems for ischemic stroke.

EV source	Animal model	Cargo loaded	Methodology of cargo loading	Encapsulation and loading efficiency (EE%/LE%)	Therapeutic outcome	Ref.
Macrophage-derived EVs	Mice/tMCAO, pMCAO	Baicalin	Sonication, incubation	45.7%/11.4%	↓ ROS by activating the Nrf2/HO-1 pathway in neurons	[96]
HEK293T cell-derived EVs	Mice and Monkeys/tMCAO, pMCAO	Circ-SCHM1	Transfection	N/A	↑ Functional recovery, brain plasticity; Inhibited glial activation, immune cells infiltration	[97]
Rat blood-derived EVs	Rats/tMCAO	Quercetin	Sonication	34%/13.6%	ROS inhibition via the Nrf/H0-1 pathway, neuro recovery	[98]
MSC-EVs	Mice/tMCAO	Curcumin	Incubation	N/A	Suppressed inflammatory response and cellular apoptosis.	[99]
Macrophage-EVs	Rats/pMCAO	Edaravone	Incubation with cells followed by EV isolation	N/A	↓ Neuronal death, shifted M1 microglia to M2 type.	[100]
M2 microglial EVs	Mice/tMCAO	NR2B9C	Sonication	29.903 ± 3.32% /18.689 ± 2.64%	Brain injury ↓ neurological deficit, P38 ↓ Neuronal survival, BCL2 ↑, Brain targeting ↑	[101, 102]
Macrophage-/MSC-EVs	Mice tMCAO/inflammation model	BDNF	Incubation, transfection	N/A, 188.8±2.92 pg in 1×10^10^ EVs	↑ Brain BDNF concentration, alleviated brain inflammation. ↑ Neuroprotection, Infarct volume ↓	[103, 104]
MSC-MNVs (Aes)	Mice/tMCAO	IONPs	Treating cells with IONPs and extrusion	N/A	↑ Anti-inflammatory response, angiogenesis, anti-apoptosis, improved targeting of ischemic brain lesion	[105]
HL-60 cell derived-NVs (Aes)	Mice/tMCAO	Resolvin D2	Sonication, incubation	10% LE, 80% drug release in 24 h	↓ Neuroinflammation ↑ Neurological functions	[106]
Embryonic stem cell-EVs	Mice/tMCAO	Curcumin	Incubation and rapid freeze-thaw cycling	N/A	↓ Inflammation, NMDAR and astrocytes expression, restored functional disabilities, ↓ infarct size, endothelial tight junction proteins in IR-mice	[107]
Mn_3_O_4_@nanoerythrocyte-T7 Aes	Mice/MCAO	Mn_3_O_4_	Treating cells with Mn_3_O_4_ and extrusion	N/A	Scavenging of free radicals and supplying oxygen before thrombolysis and suppressed oxidative stress after thrombolysis, ↑ Protection of neurocytes after MCAO	[108]
M2 macrophage-EVs	Mice/MCAO	Dnase-1	Electroporation	81.75% with loading contents of 68.125 × 10^−9^ U/particle	↓ Infarct volume, ↑ rCBF, long term neurological functional outcome, BBB integrity	[93]

### Genetically loading EVs

The traditional and most common method is genetically modifying EV-producing cells to overexpress specific molecules (proteins, miRNA, siRNA or enzymes) that are naturally incorporated into EVs during biogenesis. Genetically engineered neuron-targeting EVs were produced and utilized for the delivery of different RNAs to the brain for stroke therapy [110–113]. Rabies virus glycoprotein-circSCMH1-extracellular vesicles were developed to selectively deliver circular RNA SCMH1 to the brain. The results showed high expression of circSCMH1 in the brains of both mice and monkeys, which in turn increased functional recovery, brain plasticity and inhibited glial activation and immune cell infiltration in the peri-infarct cortex. They concluded that circSCMH-loaded EVs have the potential to increase the therapeutic window of stroke if translated to clinical practice [97].

MiR-17-92 transfected MSC-EVs were evaluated for ischemic stroke recovery. Compared with liposome treatment, the EVs group showed significant improvement in functional and behavioral recovery. These outcomes were mainly attributed to EVs ability to enhance oligodendrogenesis, neurogenesis and neural plasticity, along with the mitigation of multiple proteins in the ischemic zone [73]. In addition, miR-126-overexpressing EVs were therapeutically effective for ischemic stroke recovery by attenuating acute injury, promoting neurogenesis, inhibiting neuroinflammation and reducing infarct size [114, 115]. The Cre recombinase protein was loaded into EVs by attaching a special WW tag to the protein. This tag allows Ndfip1 to recognize and bind to the protein, making it suitable for inclusion in EVs through a process called ubiquitination. These EVs were able to deliver the protein to neurons in different brain regions [116]. Through lentiviral transfection microRNA-27a-enriched EVs derived from cerebral endothelial cells were produced and studied for enhancing axonal growth and neurological recovery after ischemic stroke. Experimental results showed that EVs preferentially localized to the presynaptic location and decreased the level of inhibitory proteins supporting axon and spine growth in injured brain regions, thus, improving neurological outcomes [117]. Through the genetic engineering of exosome-producing cell, a system named MAPLEX was developed. The idea is to attach a photocleavable protein between a cargo protein and an exosomal membrane marker. When EVs reach the target cell and are exposed to 405 nm light, the photocleavable protein is cleaved and the cargo is released to function effectively within the cell. This is a versatile strategy and could be optimized for delivery of cargo to ischemic brain regions [118]. Recently, targeted protein degradation via lysosome-targeting chimeras has emerged as a promising technology [119]. Through genetic engineering, lysosome targeting EVs named LYTEXs were generated, which can bind to specific proteins on the surface of cells and direct them to the lysosome for degradation. The LYTEX approach allows precise and efficient removal of extracellular and membrane disease-associated proteins and can be applied to the treatment and diagnosis of different ailments [120]. Genetic loading of EVs is transformative and precise in obtaining desired cargo loaded EVs, however, it is costly, time-consuming and technically complex.

### Biochemically loading EVs

Biochemical loading of EVs is accomplished either by incubating cargo with EVs or by chemically attaching it to the functional groups of EVs. Compared to genetic loading, this strategy is simpler, faster and more effective for directly loading EVs without altering parental cells. Click chemistry is frequently employed to attach therapeutic or targeting ligands to the surface of EVs. Due to its excellent biocompatibility and minimal affinity for serum proteins, dibenzocyclooctyne (DBCO) specifically reacts with azide groups, making these reagents widely used for the biochemical conjugation of therapeutic or targeting ligands to EVs. Initially, EVs were functionalized with DBCO via incubation. The therapeutic agents were then modified with azide groups. Following this, the therapeutic agents were conjugated to the surface of the EVs through a click reaction between DBCO and azide groups, facilitating simple and efficient conjugation. To enhance the brain-targeting potential of EVs, click chemistry was applied to decorate their surface with RVG29. The click conjugation of RVG29 to EVs that were loaded with cargo for injured brain therapy significantly improved their brain targeting and biodistribution profile [101, 102]. Click reactions were also used to conjugate stromal cell-derived factor-1α and the damaged blood vessel ligand DA7R to the surface of EVs. These modified EVs demonstrated improved targeting of the ischemic zone, recruitment of neural NSCs and enhanced neurogenesis [121]. Another study functionalized EV surfaces for targeting purposes with the c(RGDyK) peptide using the crosslinker DBCO [99]. However, the c(RGDyK) peptide targets αvβ3-integrin, which is overexpressed on tumor endothelial cells; therefore, EV functionalization with c(RGDyK) may not be specific to ischemic neurons [122].

MMPs especially MMP-2 and MMP-9 are overexpressed in many diseases (cancer, cardiovascular diseases, nervous system disorders, inflammation) compared to normal tissue [123]. This difference provides an opportunity to design MMP-responsive drug delivery systems, whose payload release, activation or targeting depends on MMP-mediated proteolytic cleavage.

Engineered apoptotic vesicle were biochemically decorated with an MMP-activatable cell-penetrating peptide, and their systemic injection preferentially accumulated in the ischemic brain and improved functional and histological outcomes [124]. This study suggests that MMP-responsive EVs cargo delivery is feasible in stroke therapy. However, the authors did not employ a selective MMP inhibitor to prove that proteolysis is required for targeting. Thus, EVs homing to ischemic penumbra via MMP manner requires further validation.

Click reactions are highly specific and mild, making them suitable for loading EVs without altering their structure or impairing their internalization. This ensures the safety and functionality of EVs, making click chemistry a promising tool for EV surface functionalization and cargo loading. The primary shortcoming of this approach is the low recovery of functionalized EVs following the various centrifugation steps involved in the process.

Simple incubation has also been reported for the loading of cargo into EVs before isolation or after isolation. The incubation process leverages natural electrostatic and polysaccharide interactions of EVs with the cargo. The method is straightforward, simple and depends solely on the natural affinities between the EVs and cargo. For instance, curcumin was loaded into EVs by mixing and incubating at room temperature, followed by freeze-thaw cycling. Curcumin-loaded EVs mitigated neuroinflammation, ROS, NMDAR and astrocyte expression and restored functional deficits, infarct volume and endothelial tight junction proteins after tMCAO in mice [99, 107, 125].

Edaravone protects the ischemic brain through free radical scavengers and attenuates tissue damage, delays neuronal death and improves neuronal conditions due to stroke. Due to poor availability and a short half-life, edaravone must be injected in higher doses for clinical practice. Through EVs producing cells edaravone was loaded into EVs to address its shortcomings and increase its overall stroke therapeutic potential [100]. Another group loaded BDNF protein into EVs, where bonding with BDNF was formed through interaction of the negatively charged EVs by electrostatic and polysaccharide interactions [103]. The loading efficiency depends on the hydrophobicity or charge of the cargo. Simple incubation results in low drug loading efficiency, which is the main shortcoming of this methodology.

### Physically loading EVs

Physical cargo loading of EVs involves physical or mechanical methods to temporarily permeabilize the EV membrane, allowing cargo to enter the EV core. This approach is considered one of the most efficient methods of encapsulating cargo within the EVs lumen. Common physical loading techniques include sonication, electroporation, incubation with membrane permeabilizers, extrusion and freeze-thaw cycles.

Numerous studies have explored physical loading of EVs to enhance the therapeutic outcomes of ischemic stroke. For instance, Baicalin, a potent free radical scavenger, was loaded into EVs using sonication to improve its solubility and brain-targeting capabilities. EVs significantly enhanced brain bioavailability and alleviate stroke injury by activating the Nrf2/HO-1 pathway in neurons. Furthermore, baicalin loaded EVs demonstrated superior neuroprotection and sustained release after MCAO [96]. Comparatively, baicalin loaded into liposome nanocarrier had limited neuroprotective effect and brain availability in ischemic stroke model [126]. To achieve selective targeting of neurons in ischemic brain EVs surface was functionalized with monoclonal antibody against GAP43 and loaded with quercetin through sonication. This approach increased quercetin brain accumulation by 1.7-fold and specifically targeted impaired neurons, recovering brain functionality [98].

Electroporation was used to load EVs therapeutic microRNA by applying a short electrical pulse, creating temporary pores in the EVs membrane. EVs aggregation is the common shortcoming of electroporation, which was addressed by adding trehalose to enhance the stability. This method facilitated efficient miRNA uptake, as confirmed by qPCR. These loaded EVs increased expression of microRNA within 6 h in the neurovascular unit and downregulated stroke pathogenesis genes. EVs also reduced cell death in cell lines and primary neurons after OGD and in rat stroke models [127]. CD206+ macrophage derived EVs system was developed to deliver DNase 1 to the brain for ischemic stroke therapy. DNase 1 was electroporated with EVs for 30 min, resulting in 81.75% of DNase-1 loading efficiency and a 2.3-fold increase in protein contents. This nanoformulation increased neurological recovery and promoted BBB remodeling [93]. Another study used the same source of EVs for delivery of BDNF to the brain, stating that there is no need for macrophage-derived EVs functionalization as they have native brain targeting abilities [103]. Cargo could also be loaded within EVs using a membrane permeabilizer, like saponin. A previous study showed that saponin increased hydrophilic drug loading into EVs 11-fold compared to simple incubation [128]. However, saponin concentration should be limited, as it may cause hemolytic activities. During freeze-thaw loading, cargo is mixed with EVs at room temperature for a specific duration and then rapidly frozen at −80°C or in liquid nitrogen and thawed back at room temperature, repeating the process at least three times. This method has lower drug loading and may cause EVs aggregation. In the extrusion method of cargo loading, the mixture of cargo and EVs is passed through a 100–400 nm porous membrane, which disrupts the EVs membrane and allows cargo loading within the EVs core [129].

The effectiveness of techniques for cargo loading into EVs varies depending on the nature of cargo and the source of EVs. However, methods such as sonication, electroporation and extrusion have demonstrated superior loading efficiency compared to approaches such as freeze-thawing and incubation. The main shortcoming of physical cargo loading approaches is that they might compromise the structural and functional integrity of both EVs and cargo.

Numerous studies have reported EVs-based targeted ischemic stroke therapy, however, a critical limitation of some studies lies in the lack of appropriate experimental controls to distinguish active targeting from passive targeting. EV are often modified with surface peptides, antibodies or other dendritic cell binding ligands to enhance accumulation at ischemic or immune active regions; however, few incorporate proper controls which are necessary to confirm that observed effects were from active targeting rather than passive localization via stroke induced BBB permeability. Khan *et al*. incorporated controls using naïve EVs and demonstrated superior targeting efficiency only when RVG29 ligand was present [101]. Likewise injured vascular targeting peptide DA7R and stem cell recruiting factor SDF-1 decorated dual EVs had better therapeutic effects compared to unmodified control EVs [121]. Similarly, dendritic cell derived EVs had better immune cells targeting than normal control [130]. In contrast, lactobacillus reuteri EVs were used for ischemic stroke therapy based on their intrinsic peptidoglycan mediated binding to Toll-like receptor 2. The study demonstrated improved targeting and ROS-scavenging effects, it lacks appropriate controls, making it difficult to conclude whether the targeting was truly ligand-mediated or due to passive diffusion [131]. Other studies using ligand EVs for ischemic stroke or systemic delivery demonstrated therapeutic efficiency but lacked appropriate ligand free controls [132, 133]. This highlights the need for standardized control design including EVs without targeting motifs to verify the mechanistic specificity of ligand-based active targeting strategies.

## EVs-based diagnostic systems for ischemic stroke

Biomarkers are measurable indicators present in blood, body fluids or tissues that can predict physiological or disease states, increased disease risk or pharmacological responses to therapeutic intervention. These biomarkers can be diagnostic, prognostic or predictive. In the case of ischemic stroke, a timely response is the best possible way to prevent serious complications; therefore, early and accurate diagnosis is essential for effective management, prevention and treatment.

Currently, the gold standard for stroke diagnosis involves brain imaging techniques such as magnetic resonance imaging (MRI) and computed tomography (CT). While these tools are highly effective in detecting stroke infarction, they do not provide insights into the underlying mechanisms or disease process. This limitation, coupled with the complex pathophysiology of stroke and its overlapping signs and symptoms with other neurological and non-neurological conditions, poses significant challenges for the development of highly sensitive and specific diagnostic tools [134]. EVs have emerged as important mediators of cellular communication, facilitating interactions between nearby and distant cells. During the process of EV release from brain cells, various cellular contents including nucleic acids, proteins, lipids and metabolites are secreted into EVs in a disease-specific manner. These encapsulated molecules reflect the physiological and pathological status of their parent cells, making EVs a promising tool for noninvasive diagnostic applications. For instance, EV protein and miRNA profiles have been extensively explored as diagnostic, prognostic and therapeutic targets for ischemic stroke (Table 3).

**Table 3:** EVs-based diagnostic systems for ischemic stroke.

Investigation molecule	EV source	Study model	Purpose	Mian findings/outcome	Ref.
EVs surface antigens	Human blood serum	40 TIA patients/20 controls	Diagnostic/prognostic	↓ EVs diameter and ↑EV concentration (≤150 nm and >150 nm); 10 surface antigens (CD31, CD42a, CD14, CD8, CD2, CD62P, CSPG4, CD44, CD326 and CD142) corelate with TIA.	[135]
EVs proteins	Human blood serum	571 ischemic stroke patients/controls	Diagnostic/Therapeutic	CD31+, CD144+, CD146+ and CD45+ EVs associate with stroke	[136]
EVs proteins	Human blood serum	16 ischemic stroke patients/80 controls	Diagnostic	ASC protein identified as potential biomarker of stroke	[137]
EVs proteins	Human blood serum	38 acute ischemic stroke patients/healthy controls	Diagnostic/prognostic	↑ C-reactive protein and EV number	[85]
EVs proteins	Human blood serum	10 ischemic stroke patients and 10 healthy controls	Diagnostic/Therapeutic	↑ Complement C1q subcomponent subunit B, Alpha-2-macroglobulin, Complement C1r subcomponent, Histidine-rich glycoprotein expression	[138]
EVs proteins	Human blood plasma	TIA (21) AIS (66) and controls (24)	Diagnostic	↑ CD45+, CD66+, CD235+, CD62P+ and ↓ CD235a+ EVs	[139]
EVs miRNAs (humans and mouse)	Human and mice blood	40 IS patients/33 healthy controls, mice n = 3-6 per group	Diagnostic	↑ Five humans EVs-miRNAs (hsa-miR-9-3p, hsa-miR-124-3p, hsa-miR-143-3p, hsa-miR-98-5p and hsa-miR-93-5p) and four mouse-miRNA (mmu-miR-9-5p, mmu-miR-124-3p, mmu-miR-129-5p and mmu-miR-433-3p) showed altered expression	[140]
EVs miRNAs	Rat plasma and cerebrospinal fluid	28 PE+tMCAO, Sed+tMCAO and 6 PE/Sed controls	Diagnostic/Therapeutic	↑ miRNA-92b and -370 and ↓ miRNA-136, -665, -3068 both in PE vs Sed and PE+tMCAO vs Sed+tMCAO	[141]
EVs miR233	Human blood serum	50 ischemic stroke patients/33 controls	Diagnostic	↑ mi-RNA-233 expression associated with stroke severity, occurrence and short-term outcome.	[142]
EVs miR134	Human blood serum	50 stroke patients/50 healthy controls	Diagnostic	↑ miRNA-134, IL-6 and plasma high-sensitivity C-related protein in IS patients	[143]
EVs miR-9 and -24	Human blood serum	65 stroke patients/66 controls	Diagnostic	↑ miRNA-9 and -24 corelated with severity of stroke in IS patients	[144]
EVs miR-422a and miR-125b-2-3p	Human blood plasma	55 ischemic stroke patients/55 healthy controls	Diagnostic	↓ miR-422a and -125 b-2-3p in AIS; ↑ miR-422a expression in AIS	[145]
EVs miR-21-5p and -30a-5p	Human blood plasma	143 stroke patients/24 controls	Diagnostic	↑ miRNA21-5p and -30a-5p in HIS patients compared to AIS	[146]

**Figure 4:**
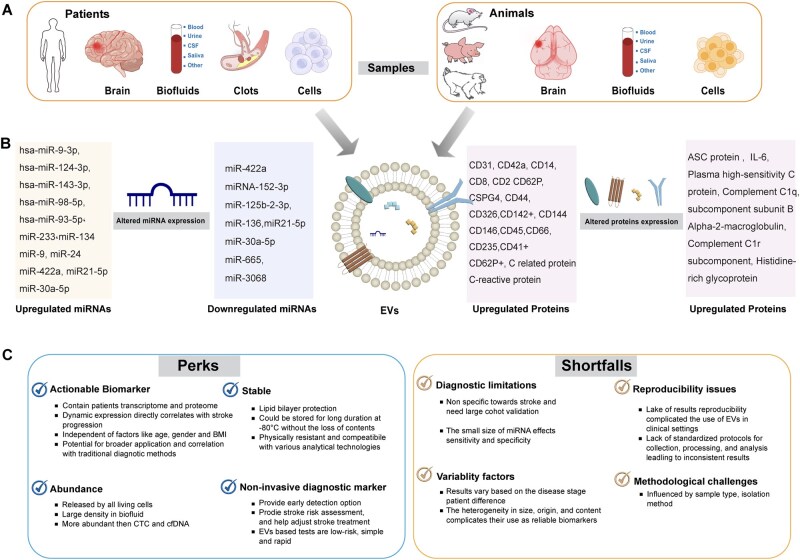
EVs as a diagnostic system for ischemic stroke. (**A**) Abundant source of EVs samples collection for diagnosis and prognosis. (**B**) EVs miRNA and proteins altered profile after ischemic stroke. (**C**) The perks and shortfalls of EVs usage as biomarker of ischemic stroke.

Changes in EV profiles have been shown to correlate with improved outcomes in clinical trials, such as the aspirin and rivaroxaban combination therapy trial for cardiovascular patients, where alterations in EV concentration and content were found to serve as potential biomarkers [147]. Ongoing research is focused on identifying stroke biomarkers that are not only specific and sensitive but also rapid, cost-effective and clinically applicable. EVs offer significant promise as diagnostic systems in this regard, as their dynamic protein and miRNA profiles can provide comprehensive insights into stroke pathophysiology, disease progression and therapeutic responses (Figure 4).

### EVs protein signature for ischemic stroke diagnosis

The protein signature of EVs offers a noninvasive and reliable diagnostic avenue, reflecting the physiological and pathological states of cells affected by ischemic stroke. For example, Burrello *et al*. [135] investigated the surface antigen profile of EVs in a cohort of patients with suspected transient ischemic attack (TIA) compared to healthy controls from various stroke centers. The study included 40 patients with suspected TIA symptoms and 20 healthy controls. Based on a precise diagnostic score (PREDISC) and diffusion-weighted magnetic resonance imaging, 28 subjects were stratified as TIA patients. Serum EVs were analyzed using nanoparticle tracking analysis and surface antigen profiling through bead capture, followed by multiplex flow cytometry. The study observed an increased concentration and reduced diameter of EVs in patients classified as very likely or probable TIA compared with controls. Specifically, EVs with a diameter of 30–150 nm versus 151–500 nm were changed. Among the 37 EV surface markers analyzed, 10 antigens (CD31, CD42a, CD14, CD8, CD2, CD62P, CSPG4, CD44, CD326 and CD142) were progressively elevated in TIA patients. Notably, CD31 (endothelial cell marker), CD42a (platelet marker) and CD14 (monocyte/macrophage marker) differentiated patients with diffusion-weighted imaging lesions from those with no lesion. While the study aligns with prior research [148, 149] and advances the potential of EVs biomarkers for ischemic stroke diagnosis, limitations include the small and potentially biased sample size and multicenter variability, which reduce generalizability. In a cohort of 571 ischemic stroke patients, elevated levels of EVs endothelium-derived markers (CD31+, CD144+ and CD146+) and leukocyte-derived markers (CD45+) in plasma were associated with ischemic stroke [136]. Similarly, an association between CD62E+ EVs levels and greater neurological disabilities and larger infarct volume has been previously reported [149]. Inflammasome-associated proteins, which are crucial in stroke pathogenesis were also investigated in circulating EVs from stroke patients and healthy controls. Among inflammatory proteins studied, interleukins (IL)-1β, IL-18, caspase-1 and apoptosis associated speck-like protein containing a caspase recruitment domain (ASC), only ASC levels were significantly elevated in stroke patients [137]. Proteomic analysis of 38 acute ischemic stroke patients revealed increased levels of inflammatory and acute-phase proteins, including C-reactive protein. Moreover, elevated EVs counts and increased mRNA expression of tumor necrosis factor, CXCL-1, IL-1β and CCL-2 were detected in active macrophage cell lines [85]. In a separate serum EVs proteomic study involving 10 ischemic stroke patients and 10 matched controls, 29 proteins were identified, with complement C1q subcomponent subunit B, alpha-2-macroglobulin, complement C1r subcomponent and histidine-rich glycoprotein upregulated in stroke patients [138]. However, the limited sample size, potential bias and restricted clinical annotation hinder the application of these findings as reliable diagnostic or therapeutic targets.

In a clinical trial [NCT01954797], dynamic changes in EVs levels were associated with functional recovery and vascular events in subacute stroke patients over 6 months. Higher levels of neuronal- and leukocyte-derived vesicles are correlated with poorer functional recovery, as assessed by the Barthel Index [150]. Agouni *et al*. [139] reported increased endothelial EVs (CD146^+^ CD62E^+^) within 48 h of transient and ischemic stroke, with further elevation in leukocytes (CD45^+^), granulocytes (CD66^+^), erythrocytes (CD235^+^) and activated platelets (CD62P^+^) EVs. Over 5- to 30-days, erythrocyte (CD235a+) EVs decreased while activated endothelial cells, leukocytes granulocytes and platelets EVs remained elevated. Advanced techniques such as proteomics, NTA and flow cytometry have revealed significant alterations in the circulating EVs profile, inflammatory proteins and complement factors in rivaroxaban-treated patients compared to controls [151].

While EVs protein profiles hold promise as biomarkers for stroke diagnosis and prognosis, large-scale, unbiased and longitudinal studies are needed to translate these findings from research to clinical practice. Standardized protocols for EVs biomarker validation are critical to ensure reproducibility and reliability across diverse populations.

### EVs miRNA profile for ischemic stroke diagnosis

EVs encapsulate miRNAs within their core, providing a protective environment that shields them from enzymatic degradation and immune clearance. This unique feature makes EVs-associated miRNAs more reliable and stable biomarkers compared to free miRNAs present in body fluids. Moreover, EVs play a pivotal role in intercellular communication, serving as carriers of molecular cargo between cells both in healthy and diseased states. Through this exchange their miRNA contents are dynamically distributed across distant sites or organs, reflecting the physiological and pathological state of the host. In the context of stroke, EV miRNAs have shown significant diagnostic and therapeutic potential, demonstrating strong association with stroke pathology. A comprehensive study examining the EV-miRNA profile as a biomarker of stroke was conducted using serum EVs from both humans and mouse models. The analysis revealed significant alterations in the expression of 424 miRNAs in ischemic stroke patients and 37 miRNAs in tMCAO mice, out of a total 1444 human and 1373 mouse serum EVs-miRNAs identified. Further validation highlighted the top five highly expressed miRNAs in ischemic stroke patients, including hsa-miR-9-3p, hsa-miR-124-3p, hsa-miR-143-3p, hsa-miR-98-5p and hsa-miR-93-5p. Similarly, in the mouse serum EVs, four miRNAs (mmu-miR-9-5p, mmu-miR-124-3p, mmu-miR-129-5p and mmu-miR-433-3p) showed a strong association with ischemic stroke (IS) injury. Their expression patterns revealed an increase shortly after ischemia onset (0.5 day), peaking at Days 1 and 3 and returning to baseline levels by Days 7 and 14. These miRNAs are either brain-specific or brain-enriched, emphasizing their relevance in stroke pathology [140]. In a bioinformatics analysis of EV-miRNA profiles following physical exercise, 41 differentially expressed miRNAs were identified in male Sprague–Dawley rats. The rats were assigned to four groups: (i) physical exercise (PE) with tMCAO (*n* = 28), (ii) PE without tMCAO (*n* = 6), sedentary (Sed) with tMCAO (*n* = 28) and Sed without tMCAO (*n* = 6). After 28 days of physical exercise, 16 miRNAs were upregulated and 25 were downregulated in the PE and PE+tMCAO groups compared to the Sed and Sed+tMCAO controls. Specifically, miRNA-92b and miRNA-370 were significantly upregulated, whereas miRNA-136, -665 and-3068 were significantly downregulated in both PE vs. Sed and PE+tMCAO vs. Sed+tMCAO groups. To understand the biological role of these differentially expressed miRNAs, the Kyoto Encyclopedia of Genes and Genome database was used. The analysis revealed that all the altered miRNAs were related to stroke pathology and had a role in post-stroke recovery, highlighting their therapeutic and diagnostic potential [141].

EV-miRNA-223 expression in ischemic stroke patients was significantly higher than that in controls, showing a positive correlation with the National Institutes of Health Stroke Scale (NIHSS) score. Elevated levels of miR-233 are associated with ischemic stroke occurrence, severity and short-term progression, underscoring its potential as a diagnostic biomarker [142]. Additionally, EVs-miR-123 expression was found to be increased within 24 h of stroke onset in the acute ischemic stroke group compared to healthy controls, as revealed by RT-qPCR analysis. This elevated mi-123 expression was strongly correlated with worse NIHSS score, larger infarct volume and poor stroke prognosis. Furthermore, miR-123 levels showed a positive correlation with increased expression of IL-6 and plasma high-sensitivity C-related protein in IS patients, suggesting miR-123 as a potential biomarker of stroke diagnosis and prognosis [143]. Another study found that EVs-miR-9 and -24 were upregulated in IS patients, with their levels positively correlating with stroke severity [144]. Similarly decreased expression of EVs-miR-152-3p was associated with increased stroke severity, indicating its potential as a biomarker for evaluating stroke progression. For early-stage diagnosis of ischemic stroke, the expression profile of EVs-miR-152-3p and -30a-5p was evaluated across five different phases: (i) Hyperacute phase (HIS, within 6 h), (ii) Acute phase (AIS, 1–3 and 4–7 days), (iii) Subacute phase (SIS, 8–14 days) and (iv) Recovery phase (RIS, *>*14 days). The plasma EVs levels of miRNA-30a-5p in the SIS and RIS groups were significantly different from those in the control group (*P* < 0.05, *P* < 0.01, respectively). In HIS group (*P* < 0.05), an increase in miR-30a-5p expression was observed, whereas a decrease was noted in the AIS group (*P* < 0.05). In AIS, both miR-21-5p and miR-30a-5p levels were reduced compared to those in the HIS group, suggesting that the severity of stroke is directly closely linked to the dynamic expression profile of these miRNAs [146]. The predictive potential of EVs miR-422a and miR-125b-2-3p was evaluated in a study involving 55 ischemic stroke patients and matched healthy controls. Patients were further categorized into the AIS and SIS phases. qRT-PCR analysis revealed a distinct expression pattern: miR-422a expression was significantly increased in AIS patients but decreased in SIS patients compared to controls, whereas miR-125b-2-3p showed reduced expression in the SIS phase [145].

Although various types of EV-associated miRNAs have demonstrated promising potential for stroke diagnosis, several challenges remain (summarized in Figure 4), including the heterogeneous cellular origins, limited stroke specificity and poor reproducibility across studies, which collectively limit their immediate clinical translation. Despite these limitations, EV-based biomarkers hold significant promise for the future. They may play a crucial role in the discovery of novel drugs for ischemic stroke [152] enable more precise disease monitoring [153] and support guided treatment decisions in upcoming clinical trials [154, 155].

## EVs-based synergistic systems for ischemic stroke

**Figure 5:**
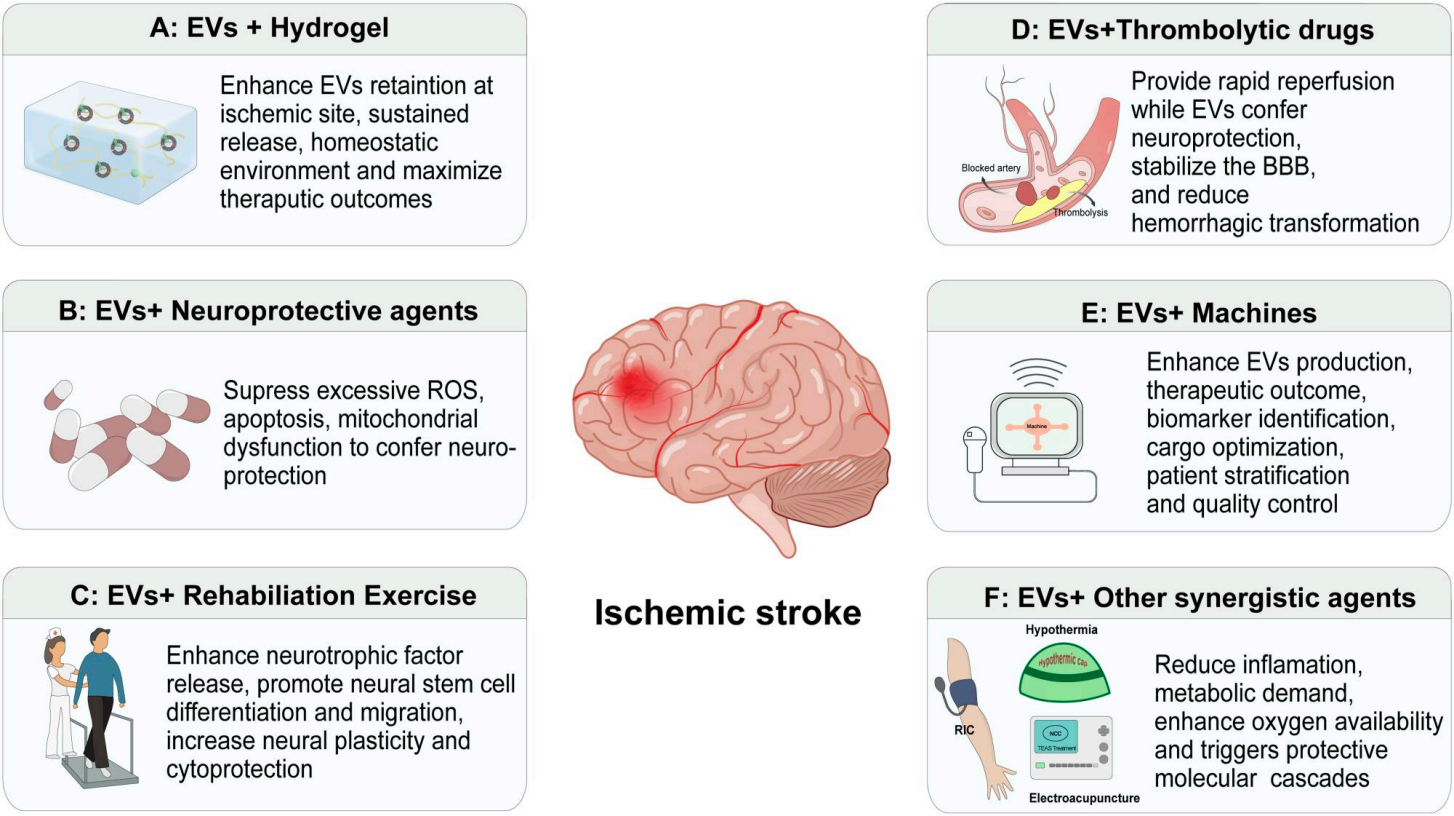
EV-based synergistic systems for ischemic stroke: (**A**) EVs in combination of hydrogel for the treatment of ischemic stroke, where the hydrogel provide physical support for the damaged tissue and allows controlled and sustained release of EVs at the site of injury. (**B**) Combining EVs with neuroprotective agents. (**C**) EVs in combination with physical exercise targeting a broader array of biological pathways and mechanisms to promote ischemic stroke recovery (**D**) Synergistic integration of EVs with reperfusion therapy, in which EVs serve as neuroprotective agents, extending the therapeutic window of reperfusion therapy as well as reducing the risk of hemorrhagic transformation. (**E**) EVs combined with machines to enhance EVs quality control, brain targeting, BBB penetration and therapeutic efficacy for ischemic stroke (**F**) EVs in combination with other potential synergistic agents for ischemic brain injury recovery.

The complex pathophysiology of stroke creates a need for the development of synergistic systems that can target multiple pathways, as therapies targeting a single pathway often fall short to provide sufficient cytoprotection. Researchers are actively exploring synergistic systems to improve the therapeutic efficacy of EVs in the treatment of ischemic stroke. The combination of EVs with other therapeutic agents may offer a more potent and comprehensive solution, leading to improved functional recovery and better clinical outcomes for brain ischemia. In this section, we provide a detailed overview of these systems and explore their potential applications and advantages and highlight how the integrated use of EVs and other therapeutic agents could enhance ischemic stroke recovery (Figure 5).

### Hydrogel

EVs efficient retention, controlled release and integration in the target lesion site is of utmost importance for maximizing therapeutic outcome in CNS disorders. The most suitable agent that can overcome this shortcoming of EVs is the use of hydrogels. Loading EVs into a hydrogel can prevent their premature clearance and allow localized and concentrated delivery to the target lesion by placing the EV-loaded hydrogel right at or in the vicinity of the target site [156]. Hydrogels have favorable safety profile, broad biomedical applications and high biocompatibility with brain tissue, while enabling targeted and sustained release of EVs [157, 158]. Hydrogel has been assessed for the treatment of deep irregular brain lesions in rat stroke models, which makes them attractive for treating brain disorders [159]. Hyaluronic acid-based hydrogels have been utilized for the sustained release of NSC-EVs to promote ischemic stroke recovery. The mouse tMCAO model was used to demonstrate the therapeutic outcome of hydrogel-loaded EVs in comparison with the control treatments. EV-hydrogel therapy reduced infarct volume, enhanced cerebral angiogenesis and improved neurological function [160].

This study demonstrated that a hydrogel-based controlled release system could be a promising approach for enhancing angiogenesis and recovery after stroke. Previous studies have also demonstrated the effectiveness of hydrogel-based localized and sustained drug release systems for ischemic stroke. Multiparameter hydrogels were prepared to optimize EVs/drug release, storage modulus and degradation profile. Dual-function hydrogel capable of sequential release of two different therapeutics were engineered by varying the polymer composition and cross-linking ratio, which allowed tuning of the network density and degradation kinetics so that small molecules and protein cargos exhibited distinct, temporally separated release profiles [161]. Similarly, an injectable, brain-compatible hydrogel was prepared, in which mechanical properties and gelation kinetics were systematically adjusted to enhance injectability, retention at the lesion site and sustained drug release over weeks [162]. Fan *et al*. developed an electroconductive, EV-loaded hydrogel for spinal cord repair and precisely tuned cross-linked conductive polymers to regulate hydrogel stiffness, degradation rate and EV release behavior, more conductivity and density cross-linked formulation slowed EVs release and enhanced retention. Softer networks facilitated faster vesicle diffusion and earlier immunomodulatory effects [163]. Natural extracellular matrix (ECM) bioscaffold inherently contain matrix-bound nanovesicle embedded within biochemical and biomechanical landscape shaped by the ECM’s own cross-linking and stiffness characteristics, providing strong evidence that EV release and retention kinetics are influenced by matrix architecture. The combination of the ECM and EVs maintained tissue homeostasis and neuroblastoma cell differentiation. This synergistic system leverages the mechanical support of the scaffold and the biological signaling capabilities of the EVs, optimizing the healing environment and making it more conducive for ischemic recovery and cellular regeneration [164]. In addition to EV retention and release kinetics, hydrogel-based system can modulate post-stroke tissue mechanics, which regulate astrocyte transformation, glial scar stiffness and neuroregeneration via Piezo1-Wnt7b signaling pathway [165]. These studies demonstrate that iterative tuning of hydrogel parameters optimize EVs/drug release and highlight the importance of design-of-experiments-style formulation refinement, which is largely absent in current stroke-related EV-hydrogel literature.

EVs released from MSC cultured on 3D-GelMa hydrogels were more effective than their counterpart in brain inflammation mitigation after stroke. The 3D-derived EVs reduced neuronal death and shifted inflammatory microglia to protective state in mouse ischemia-reperfusion injury models. This study demonstrated the biocompatibility of hydrogels for MSCs and the enhanced neuroprotective function of their EVs [166]. Recently, an innovative implantation strategy was developed using human MSC-derived EVs immobilized in a peptide-modified adhesive hydrogel, which provides exosome-encapsulated extracellular matrix to injured tissue. Implantation therapy effectively promoted nerve recovery and motor function in rats, suggesting that EVs implanted in hydrogels can be an effective treatment for CNS disorders [167]. Hydrogels loaded with EVs for nerve repair, mitigation of oxidative stress and inflammation had promising results after spinal cord injury compared to systemically administered EVs [168–170]. The EVs-loaded hydrogel had better cardiac function recovery in a rat model of myocardial infarction *in vivo* after 4 weeks, compared with free EVs [171]. EVs encapsulated in hydrogels represent a promising and clinically efficient approach for stroke therapy. Importantly, a methodological gap exists in studies utilizing hydrogel systems for EVs delivery in ischemic stroke. When hydrogel is used to deliver EVs in stroke therapy, claims of sustained or responsive release are common, but most studies lack an inert or noncleavable hydrogel controls to confirm whether benefits arise from controlled EVs release or from passive diffusion. For example, Gu *et al*. [160] demonstrated enhanced angiogenesis using an adhesive EVs-hydrogel but lacked a nondegradable control and Tan *et al*. [172] reported improved tissue retention without testing passive diffusion controls. In contrast, Cao *et al*. [173] incorporated both ROS-responsive and nonresponsive hydrogels, confirming that therapeutic efficacy depended on stimulus-triggered EV release. Future studies should incorporate noncleavable hydrogel controls and EVs-free hydrogel to clearly distinguish material-driven effects from EV-mediated therapeutic outcomes in EV-hydrogel systems.

### Thrombolytic and neuroprotective agents

EVs could be used in combination with neuroprotective and thrombolytic agents to enhance treatment efficacy in ischemic stroke therapy. The thrombolytic agent tPA is the gold standard for ischemic stroke; however, hemorrhage transformation following BBB disruption from tPA therapy is a critical issue. EVs therapy has been shown to alleviate tPA induced hemorrhage by inhibiting astrocytes activation and inflammation and maintaining BBB integrity [80]. The therapeutic potential of cerebral endothelial cell-derived EVs combined with tPA was explored and co-administration significantly reduced infarct size, improved cerebral blood flow and minimized BBB leakage in both embolic and tMCAO rodent models. Moreover, EVs address the complications associated with tPA therapy, such as BBB disruption, by stabilizing the vasculature and decreasing thrombosis-related proteins [174].

EVs in combination with rosuvastatin were administered for 7 days after MCAO in rats. The combination therapy remarkably reduced the infarct volume, which was linked to reduced level of NLP-1 and NLP-3 genes. The brain inflammation maker GFAP-positive cells and lipid peroxidation marker, malondialdehyde levels were reduced compared to single-treatment groups [175]. Although the ongoing clinical trial did not produce the expected outcome in the combination therapy of neuroprotectant and thrombolysis, researchers are actively attempting to address these limitations. We propose that improved outcomes might be achieved if such trials were conducted in combination with EVs. The neuroprotectant NR2B9C could be loaded into EVs and would perform better with tPA than when conjugated to HIV-derived TAT, as TAT exhibits off-target effects and it is sensitive to tPA-mediated degradation [176, 177]. In a mouse myocardial ischemia/reperfusion model, the combination of EVs with hybrid nanovesicles reduced inflammation and helped clear dead cells via the immune mechanism, similar synergistic effects were observed in autoimmune skin disorders [178, 179]. Combinational therapy using two distinct EV populations also demonstrated synergistic effects for ischemic stroke therapy in a mouse pMCAO models [180]. Neuroserpin, a potent neuroprotective agent which has the potential to extend the therapeutic window and mitigate tPA induced BBB damage and confer neuroprotection after brain ischemia. Research has confirmed the presence of Neuroserpin in EVs and in the future, it will be interesting to investigate EVs loaded with or over-expressing neuroserpin in combination with tPA to evaluate the synergistic effects [181–183]. Moreover, other neuroprotective agents have also been studied for their therapeutic efficacy via EV-based combined systems [184]. EVs have shown to enhance the therapeutic efficacy of neuroprotectants as well as they have been found to mitigate the side effects associated with thrombolytic agents. Thus, EVs-based synergistic systems with neuroprotective or thrombolytic agents offer a promising therapeutic approach that ensures protection and regeneration of the neural landscape as well as rapid reperfusion after ischemic stroke.

### Exercise and rehabilitation

Exercise is a well-established nonpharmacological intervention for vascular disorders, and for the prevention and treatment of stroke, as recommended by the American Heart Association. It has been proven clinically that being physically inactive is associated with stroke severity. Various positive effects of exercise have been explored, such as reducing stroke risk factors, enhancing the release of neurotrophic factors, promoting neural stem cell differentiation and migration and increasing neural plasticity [185]. The function of circulating endothelial cell-derived exosomes is directly related to moderate exercise, helping endothelial cells protect against hypoxic injury. Exercised mice mice had a decreased neurological deficit score, reduced infarct volume, decreased cell apoptosis and increased microvessel density in the acute stage of stroke (Day 2 after MCAO) compared to sedentary controls. The levels of exosomes and miR-126 increased both in brain tissues and plasma, which were positively correlated with exercised mice. While again at chronic stage of stroke (Day 28 after MCAO) exercised mice had reduced infarct lesions, faster angiogenesis/neurogenesis greater microvessel density and enhanced sensory motor function. An increase in the amount of BDNF, p-TrkB/TrkB and p-Akt/Akt was found in the exercised mouse brain, indicating that moderate exercise induces the release of miR-126-enriched EVs that are beneficial in MCAO-induced ischemic injury [185]. The synergistic effect of treadmill exercise and MSC-EVs for brain ischemia was also investigated. Fourteen days of combined exercise and EVs therapy reduced neuronal death, smaller infarct volume and better neurological recovery as compared to control. Mechanistically JNK1/c-Jun signaling pathway activation was responsible for these improvements [186]. In another study, rats subjected to treadmill exercise (30 min/day at up to 25 m/min) for three or more weeks exhibited neuroprotection through downregulation of the Bax/Bcl-2 ratio and modulation of caspase-3 after stroke onset. [187]. Exercise-induced increases in EVs-associated miR-342-5p were also observed following swimming exercise, leading to downregulation of Ppm1f protein expression and conferring cardioprotection [188]. Rehabilitation has been widely reported, to be effective in improving recovery in patients following ischemic stroke [189, 190]. The integration of rehabilitation with EVs therapy could further enhance stroke recovery. Studies evaluating the effects of animal handling and administration of either multiple or a single dose of EVs revealed that behavioral engagement was essential for EVs treatment efficacy at specific dose and time point. These findings emphasize the importance of combining rehabilitation strategies with EV therapy to maximize functional recovery after stroke [191]. Collectively, these studies highlight the crucial role of exercise and rehabilitation in synergy with EVs treatment to improve neurological functions and reduces brain damage.

### Machines

Integration of machines with EVs in the context of ischemic stroke could be helpful in enhancing therapeutic outcome, biomarker identification, cargo optimization, patient stratification and quality control [192]. Noninvasive diagnosis of EVs is limited because of the time-consuming isolation techniques and lack of precise tests to detect EVs contamination. By applying machine learning (ML) and advanced imaging models, EVs purity has been improved and apolipoprotein A1 has been identified as a protein marker indicative of common contamination [193]. Sensor combined with ML have also enabled noninvasive liquid biopsy approached for of EVs biomarker detection. Using this platform, the EVs signature showed 91.1% accuracy in differentiating breast cancer stages [194]. Another study demonstrated that ML algorithms can identify age-related changes in EVs nucleic acids [195]. These findings suggest that machine integration could enhance EVs quality and reliability for clinical applications. Neuromorphic-based sensing technologies, which capture intensity changes as asynchronous spikes and are ideal for studying tiny fast-moving entities such as EVs. When combined with computational platform, these systems could enable real-time tracking of EVs circulation within the brain, facilitating detailed analysis of EV. Through this combined approach, details about EVs cellular targeting, binding and repair mechanisms could be easily studied to improve clinical outcomes [196, 197]. Advanced ML algorithms could also predict therapeutic agents best suited for combination with EVs, thereby boosting their efficiency for repairing damaged tissue and supporting recovery. For instance, a high-speed experimental setup used a machine learning model to analyze millions of drugs and identified 100 drugs that could self-assemble into nanoparticles with 1.8% accuracy. These nanoparticles showed improved safety, efficacy and ease of use for drug delivery for chronic disease. Combining EVs therapy with these advanced drug delivery technologies could significantly improve targeted treatment and therapeutic outcome [198–200]. Machine-learning-based nano theranostics can further uncover complex relationships between nanomaterial features and biological performance, offering a roadmap for data-driven engineering of EVs in stroke therapy [201]. The combination of EVs and machines is promising; however, the key challenge is the need for large, reliable and well-annotated datasets to train robust and generalizable models.

For effective brain drug delivery, noninvasive low-intensity focused ultrasound is commonly utilized, which uses sound waves to potentially open up the BBB and increase the localization of i.v. administered EVs. Optimal ultrasound stimulation has been reported to improve blood flow, angiogenesis, synaptogenesis and to improve neurological outcomes after brain ischemia [202]. Ultrasound use increased the EVs concentration in the targeted right hippocampus of rats compared to the left control [203]. It stimulates astrocytes to produce 5-fold more EVs. These ultrasound-stimulated EVs were effective in reducing the harmful effects of Aβ on neurons. Researchers have also found that focused ultrasound is helpful in opening the BBB, allowing more EVs to reach the brain cells and perform therapeutic functions [204]. An EV-based synergistic system combined with electroacupuncture was developed and assessed in mouse stroke model. The combined therapy improved neurological functions, reduced brain damage and decreased cellular death in mice. This treatment also reduced pro-inflammatory T cell responses and enhanced regulatory T cell activity. Moreover, combined treatment enhanced neuroprotection via the IL-33/ST2 pathway and reduced the activation of microglia and astrocytes [205]. Another study developed a pulsed focused ultrasound strategy to guide EVs to stroke-induced injury sites, thereby enhancing brain targeting. However, the addition of gas nanobubbles in combination of high ultrasound caused adverse effects, including microhemorrhages and white matter damage, highlighting the need for careful optimization of ultrasound parameters [206].

### Other potential synergistic systems

Agents such as hypothermia, remote ischemic conditioning (RIC), electroacupuncture, artificial EVs or normobaric oxygen are being studied for their potential to optimize ischemic stroke recovery outcomes. These therapies offer unique mechanisms that could potentiate the reparative effects of EVs, from reducing metabolic demands and inflammation with hypothermia to enhancing oxygen availability with normobaric oxygen and triggering protective molecular cascades with remote ischemic conditioning. Infusion of plasma-EVs from RIC-treated mice improved neurological function and reduced infarct size compared to non-RIC-treated mice plasma EVs. RIC-derived EVs had higher levels of hypoxia-inducible factor-1 than controls. Another study demonstrated that RIC exerted neuroprotective effects and increased cellular resilience in an *in vitro* ischemic stroke model of OGD. These effects were mainly due to the release of exosomal miR-126, which lowered DNMT3B expression, a regulator of DNA methylation [207]. EVs also have the potential to work synergistically with hypothermia therapy. Intra-arterial infusion of iced lactated Ringer’s solution (0–4°C) into the MCA vessel significantly improves infarct volume [208]. Since EVs are more stable at lower temperatures, incorporation of EVs during or shortly after hypothermia treatment could enhance the therapeutic impact on damaged neurons and other cells.

Electroacupuncture (EA), a modality that combines traditional acupuncture techniques with modern electrotherapy, has been explored as a therapeutic option for the treatment of ischemic stroke. Combining EA with EVs therapy presents an innovative approach of a synergistic system that improves neurological function and accelerates recovery. Researchers have investigated the influence of EA and EVs on the microbiome gut-brain axis after stroke in mice. Mice treated with the combination therapy showed significant improvements in neurological function and reduced damage to the brain and intestines. Alterations in immune markers, decrease in IL-17 and increase in IL-10 in the brain and colon tissues were observed after stroke [209]. In another study, the combined therapy of EA and EVs improved neurological functions, decreased brain damage and reduced nerve cell death in MCAO mice. The treatment also suppressed immune responses (Th1 and Th17) while enhancing regulatory T-cell responses. Moreover, EA and EVs therapy provided neuroprotection by inhibiting the activation of microglia and astrocytes [205]. These studies suggest that EA along with EVs has synergistic effects and offers a promising new treatment for ischemic stroke.

An innovative extension of EV-based therapy with inherent synergies is Artificial EVs (AEs). AEs overcome the limitations of endogenously produced natural EVs, such as heterogeneity and low yield in cell culture. AEs can be produced in significantly higher quantities (100- to 250-fold higher than native EVs) [210–212] using physical methods such as ultrasonication, nitrogen cavitation and extrusion [105, 106, 213, 214]. AEs have the same characteristics as natural EVs in the context of therapeutic potential, cargo loading and surface functionalization abilities [215]. Recent studies have demonstrated the potential of AEs in ischemic stroke therapy. For example, AEs derived from iron oxide nanoparticle-treated MSCs exhibited high levels of therapeutic growth factors, promoting ischemic stroke recovery through enhanced anti-inflammatory response, angiogenesis and anti-apoptosis. Magnetic navigation further improved brain targeting by 5.1-fold [105]. Additionally, AEs were prepared from HL-60 cells by disrupting cells with nitrogen cavitation at a pressure of 350–400 psi, followed by centrifugation. The prepared AEs were loaded with neuroprotective resolvin D2 for the treatment of ischemic stroke. AEs delivered cargo to the brain, which was confirmed by live imaging of mouse brain through intravital microscopy. This treatment decreased neuroinflammation and improved neurological functions in the ischemic stroke mice [106]. Innovative approaches have also explored multifunctional AEs. For instance, erythrocytes treated with nanoenzyme (Mn_3_O_4)_ and a BBB crossing peptide (T7) were extruded to create AEs capable of scavenging free radicals, supplying oxygen before thrombolysis and suppressing oxygen boosts afterward. Such systems highlight the versatility of AEs in addressing stroke pathophysiology [108]. AE systems have also been designed to enable irreversible ligand binding and prolonged tissue retention [216], demonstrating how bioinspired nanovesicle engineering can enhance stability and therapeutic persistence, which could be adapted to ischemic stroke therapy.

While AEs hold great promise as next-generation EVs-based therapeutic platforms [217], challenges such as functional purity, heterogeneity and clinical-scale production must be addressed to enable their clinical translation. Despite these hurdles, AEs represent an innovative extension of EVs-based therapy, offering scalability, multifunctionality and enhanced therapeutic potential for ischemic stroke recovery.

Normobaric oxygen therapy is another potential ischemic stroke therapy which improves oxygenation, preserve the BBB integrity and reduce brain injury in stroke patients by mitigating neuroinflammation, oxidative stress and apoptosis [218, 219]. Combining normobaric oxygen therapy with EVs could enhance outcomes by addressing multiple pathological pathways simultaneously. Given the complex pathophysiology of ischemic stroke, it is crucial to explore therapeutic strategies that combine EVs with other agents that target multiple pathways simultaneously. Future research should focus on rigorous preclinical validation and clinical evaluation of these combination therapies to advance innovative, multimodal solutions for ischemic stroke management.

## Conclusion and outlook

In the treatment of ischemic stroke, EVs exhibit significant potential for modulating the ischemic environment, attenuating excitotoxicity, mitochondrial dysfunction and apoptotic cell death, while promoting angiogenesis, neurogenesis and functional recovery. Collectively, these properties endow EVs with regenerative capabilities that extend beyond those of conventional stroke therapies. The use of EVs as therapeutic agents, cargo delivery systems, biomarkers and synergistic modulators represents a rapidly expanding field with substantial translation potential. EVs have been successfully investigated for the treatment of brain pathologies, and accumulating experimental evidence highlights their effectiveness in promoting ischemic stroke recovery through multiple complementary mechanisms. Clinical translation is now underway supported by early proof-of-concept studies. However, small phase I clinical trials remain essential to address key translational challenges, including EV scalability, batch-to-batch consistency, good manufacturing practice, long-term storage stability and rigorous quality control. Several clinical trials are investigating the therapeutic and diagnostic potential of EVs in ischemic stroke (Table 4). Notably, three ongoing phase I/II clinical trials are evaluating the safety and efficacy of intravenously administered EVs for reducing post-stroke disability, promoting neurogenesis and enhancing recovery. In addition to therapeutic applications, diagnostic studies are exploring EVs as biomarkers for stroke, focusing on their potential to predict post-stroke cognitive decline, assess functional outcomes and improve the diagnosis of both transient ischemic stroke and acute ischemic stroke. Together, these trials represent pivotal steps toward integrating EVs into clinical practice, both as therapeutic agents for mitigating stroke-induced damage and as biomarkers for early diagnosis, risk stratification and monitoring recovery. While some trials have been completed, many remain ongoing and their findings are expected to substantially advance EV-based stroke management.

**Table 4:** EVs-based clinical tails for ischemic stroke.

Identifier	EVs source	Status	Location	Brief summary of the trail
NCT06138210	Human iPSC-derived EVs	Recruiting	Beijing, China	Assesses the safety and initial efficacy of intravenously administered EVs in patients with acute ischemic stroke
NCT05524506	Microparticles	Completed	Stockholm, Sweden	Investigated the biomarker of TIA or ischemic stroke
NCT03384433	MSC-derived EVs	Unknown	Tehran, Iran	Evaluates the effect of MSC-EVs on reducing neurological disability in patients with acute ischemic stroke
NCT06612710	Human-induced neural stem cell-derived EVs	Recruiting	Wuhan, Hubei, China	Evaluate the safety, tolerability and preliminary efficacy of NSC-EVs for treatment of ischemic stroke
NCT06257823	Blood-EVs	Recruiting	Aarhus, Denmark	Investigate EVs profile as predictor of post stroke cognitive decline and dementia
NCT04266639	Blood-derived EVs	Completed	Aarhus, Denmark	Explored the role of EVs as biomarkers for stroke
NCT05370105	Blood serum-derived EVs	Active, not recruiting	Florence, Italy	Uses a surface plasmon resonance imaging-based biosensor to analyze blood EVs as biomarker of stroke before and after rehabilitation protocols in stroke patients
NCT06319742	Blood-derived EVs	Recruiting	Lugano, Ticino, Switzerland	Aim to validate blood-derived EVs as biomarker to improve diagnosis and classification of TIA and ischemic stroke
NCT01954797	Neuronal and leukocyte-derived-EVs	Completed	Berlin, Germany	Investigated dynamic change in EVs populations after stroke, and their association with functional outcomes
NCT05645081	Endothelial-derived EVs	Recruiting	Merthyr Tydfil, United Kingdom	Aim to determine whether elevated endothelial EVs are associated with increased ischemic stroke risk and correlate with prolonged prothrombin time

Despite these advances, the therapeutic efficacy of EVs remains highly dependent on multiple variables, including cellular source, culturing conditions and isolation protocols. The use of homogeneous and well-characterized cell sources is crucial, as donor variability in heterologous primary cells can compromise reproducibility and therapeutic consistency. For example, MSC-EVs have been reported to promote immune tolerance or induce cytotoxic effects depending on culture conditions [31, 35]. Moreover, EVs derived from certain pathological or inflammatory cell sources have been shown to trigger neuroinflammation and ferroptosis and exacerbate neurological deficits after ischemic stroke [220, 221]. Importantly, optimal EV selection should be tailored to disease-specific therapeutic goals. MSC-EVs are particularly suited for immune modulation, whereas NSC-EVs may be more effective for enhancing neural plasticity and repair. Similarly, the route of administration should be selected based on the intended mechanism of action, with systemic delivery (e.g. i.v.) favoring immune modulation and localized delivery (e.g. intracerebroventricular) offering advantages for mitochondrial stabilization and direct neural support [35, 222]. Scalable, cost-effective production methods and standardized isolation techniques capable of yielding high-purity EVs preparations are urgently needed to support clinical.

To address the issues of low yield and purity of EVs and enhance their therapeutic potential, a PURE platform was developed. This platform increased the EVs yield 12 times and the concentration of desired therapeutic contents 146-fold compared to naive EVs [223].

Rapid EVs clearance and low accumulation at ischemic site remains major challenge. EVs loaded with therapeutics must also ensure efficient cargo release from the EV core after cellular uptake. Recently, researchers developed a photoinducible cargo protein release system termed MAPLEX, whereby blue-light irradiation triggers intracellular release of cargo protein, attached to exosomal marker. This system enabled gene regulation, recombination and target DNA editing in Alzheimer disease mouse model [118]. To enhance EVs clinical translation, it is crucial to optimize their therapeutic window, dosage, administration route and source for better therapeutic outcomes.

Research should focus on improving EVs cargo release, stability, safety, scalability and regulatory compliance to increase the precision of EV-based targeted drug delivery. EVs cargo loading methods—genetic, biochemical and physical—each have their benefits and shortfalls. Genetic loading is precise but complex and expensive, while biochemical methods offer effective control over EVs surface modifications but suffer from low EV recovery yields. Physical loading, although capable of achieving high cargo encapsulation, may damage EV-surface essential proteins and carbohydrates and typically requires specialized expertise and sophisticated equipment. Systematic optimization of these approaches will be essential for advancing EV-based targeted therapies for ischemic stroke. Advances in EV-based synergistic systems are also required to address the complex and multifactorial pathological processes that occur after ischemic brain injury. However, just as important as identifying new combined intervention to pair up with EVs, it is equally critical to understand their mechanistic basis, long term efficacy and safety profile before considering clinical applications. Long-term functional evaluations, as recommended by the Stroke Treatment Academic Industry Roundtable (STAIR) [224], should track motor and behavioral evaluations extending several months post treatment.

Despite the rapid expansion of EVs research, long-term biodistribution, clearance and histopathological safety data remain limited. Pharmacokinetics and safety investigation studies in rodents and non-human primates demonstrate EVs tolerability and early clearance kinetics, however, these studies primarily assess short term endpoints and do not adequately determine whether EVs membrane protein, lipids or cargo persist long term in tissues or whether delayed hepatic, renal or immune toxicities may occur [217, 225, 226]. EVs differ substantially in membrane composition, cargo and biogenesis route, resulting in highly variable organ tropism and metabolic fates complicating standardized toxicity assessment [227]. Single-vesicle technologies are only now becoming available, enabling more reliable detection of rare EV subpopulations that may disproportionately influence long-term biodistribution or immunogenicity. These advances offer powerful new tools to improve chronic safety evaluation [228, 229]. To date, few preparations tested in animal models have also been tested in humans. This limited testing means that it is uncertain whether the positive results observed in rodents could apply to humans. To bridge this translational gap, additional well-designed clinical trials are essential to validate the safety, effectiveness and clinical applicability of EV-based therapies and to inform future treatment guidelines. Moreover, market access, manufacturing cost and overall clinical value must be carefully considered when preparing EVs for clinical use. Currently, there is no FDA-approved EVs product, however, the field is rapidly advancing toward scalable, standardized and regulatory-compliant production frameworks to support successful clinical translation.

## Supplementary Material

rbag038_Supplementary_Data
