# Supplementary material for: Harnessing extracellular vesicles for ischemic stroke management
Source: Regen Biomater. 2026 Mar 5;13:rbag038. doi: 10.1093/rb/rbag038 (PMC13082904; doi:10.1093/rb/rbag038)
Supplement: rbag038_Supplementary_Data [file rbag038_supplementary_data.zip › 14-Mar-2026_100317_Supplementary_File.docx]

**Harnessing extracellular vesicles for ischemic stroke management**

Khan Haroon^1,2,3^, Jie Yu^2,3,6^, Renke Li^2,3,4,5*^, Chuancheng Ren^1*^, Zhaoting Li^2,3*^

^1^ Department of Neurology, The Second Affiliated Hospital, School of Medicine, The Chinese University of Hong Kong, Shenzhen & Longgang District People’s Hospital of Shenzhen, Guangdong, 518172, P.R. China

^2^ Department of Pharmaceutical Science, Division of Biomedical Health Sciences, School of Medicine, The Chinese University of Hong Kong, Shenzhen, Guangdong 518172, P.R. China

^3^ Department of Biomedical Sciences, Division of Biomedical Health Sciences, School of Medicine, The Chinese University of Hong Kong, Shenzhen, Guangdong 518172, P.R. China

^4^ Toronto General Hospital Research Institute, Division of Cardiovascular Surgery, University Health Network, Toronto, Canada

^5^ Department of Surgery, Division of Cardiovascular Surgery, University of Toronto, Toronto, Canada

^6^ NMPA Key Laboratory for Research and Evaluation of Pharmaceutical Preparations and Excipient, State Key Laboratory of Natural Medicines, Department of Pharmaceutics, China Pharmaceutical University, Nanjing, 210009, P.R. China

*Correspondence to: Renke Li, Chuancheng Ren, Zhaoting Li

E-mails: [ren-ke.li@uhn.ca](mailto:ren-ke.li@uhn.ca), [rccnsf@sina.com](mailto:rccnsf@sina.com), [lizhaoting@cuhk.edu.cn](http://lizhaoting@cuhk.edu.cn)

**Authors footnote**

**Haroon Khan** is a Postdoctoral Fellow at Department of Neurology, The Second Affiliated Hospital of The Chinese University of Hong Kong (Shenzhen)/Longgang District People’s Hospital.

Email: [Haroonkhan8888@hotmail.com](mailto:Haroonkhan8888@hotmail.com)

**Jie Yu** is a Master’s student jointly trained by China Pharmaceutical University and The Chinese University of Hong Kong (Shenzhen).

Email: [yj_01828@163.com](mailto:yj_01828@163.com)

**Professor Ren-Ke Li** is Tier 1 Canada Research Chair in Cardiac Regeneration and a Fellow of the Royal Society of Canada, the Canadian Academy of Engineering, and the Canadian Academy of Health Sciences.

Email: [ren-ke.li@uhn.ca](mailto:ren-ke.li@uhn.ca)

**Professor Chuancheng Ren** is Chief Physician, Doctoral Supervisor, and Director of the Department of Neurology at The Second Affiliated Hospital of The Chinese University of Hong Kong (Shenzhen)/Longgang District People’s Hospital.

Email: [rccnsf@sina.com](mailto:rccnsf@sina.com)

**Professor Zhaoting Li** is Principal Investigator and research group leader at the School of Medicine, The Chinese University of Hong Kong, Shenzhen. He is also a Doctoral Supervisor and a Presidential Young Scholar.

Email: [lizhaoting@cuhk.edu.cn](http://lizhaoting@cuhk.edu.cn)

**Abbreviations**

Artificial EVs: AEs

Mesenchymal stem cells: MSCs

BBB: Blood brain barrier

ROS: reactive oxygen species

OGD: Oxygen glucose deprivation

NMDAR: N-methyl-D-aspartate receptor

AMPA: α-amino-3-hydroxy-5-methyl-4-isoxazolepropionic acid receptor

TIA: transient ischemic stroke

PREDISC: Precise diagnostic score

tPA: Tissue plasminogen receptor

ASC: adipose derived stem cells

MSC: Mesenchymal stem cells

DBCO: Dibenzocyclooctyne

IL: Interleukins

ASC: Apoptosis associated speck-like protein containing a caspase recruitment domain

mNSS: Modified neurological severity score.

ESCRT: Endosomal sorting complexes required for transport

TSG101: Tumor susceptibility gene 101

ALIX : ALG2-interacting protein X

tMCAO: Transient middle cerebral artery occlusion

PE: Physical exercise

ML: Machine learning

RIC: remote ischemic conditioning

EA: Electroacupuncture
